# Cross-species reactivity of antibodies against *Plasmodium vivax* blood-stage antigens to *Plasmodium knowlesi*

**DOI:** 10.1371/journal.pntd.0008323

**Published:** 2020-06-19

**Authors:** Fauzi Muh, Namhyeok Kim, Myat Htut Nyunt, Egy Rahman Firdaus, Jin-Hee Han, Mohammad Rafiul Hoque, Seong-Kyun Lee, Ji-Hoon Park, Robert W. Moon, Yee Ling Lau, Osamu Kaneko, Eun-Taek Han

**Affiliations:** 1 Department of Medical Environmental Biology and Tropical Medicine, School of Medicine, Kangwon National University, Chuncheon, Gangwon-do, Republic of Korea; 2 Department of Medical Research, Yangon, Republic of Myanmar; 3 Department of Infection Biology, Faculty of Infectious and Tropical Diseases, London School of Hygiene and Tropical Medicine, London, United Kingdom; 4 Department of Parasitology, Faculty of Medicine, University of Malaya, Kuala Lumpur, Malaysia; 5 Department of Protozoology, Institute of Tropical Medicine (NEKKEN), Nagasaki University, Sakamoto, Nagasaki, Japan; Universidade Federal de Minas Gerais, BRAZIL

## Abstract

Malaria is caused by multiple different species of protozoan parasites, and interventions in the pre-elimination phase can lead to drastic changes in the proportion of each species causing malaria. In endemic areas, cross-reactivity may play an important role in the protection and blocking transmission. Thus, successful control of one species could lead to an increase in other parasite species. A few studies have reported cross-reactivity producing cross-immunity, but the extent of cross-reactive, particularly between closely related species, is poorly understood. *P*. *vivax* and *P*. *knowlesi* are particularly closely related species causing malaria infections in SE Asia, and whilst *P*. *vivax* cases are in decline, zoonotic *P*. *knowlesi* infections are rising in some areas. In this study, the cross-species reactivity and growth inhibition activity of *P*. *vivax* blood-stage antigen-specific antibodies against *P*. *knowlesi* parasites were investigated. Bioinformatics analysis, immunofluorescence assay, western blotting, protein microarray, and growth inhibition assay were performed to investigate the cross-reactivity. *P*. *vivax* blood-stage antigen-specific antibodies recognized the molecules located on the surface or released from apical organelles of *P*. *knowlesi* merozoites. Recombinant *P*. *vivax* and *P*. *knowlesi* proteins were also recognized by *P*. *knowlesi-* and *P*. *vivax*-infected patient antibodies, respectively. Immunoglobulin G against *P*. *vivax* antigens from both immune animals and human malaria patients inhibited the erythrocyte invasion by *P*. *knowlesi*. This study demonstrates that there is extensive cross-reactivity between antibodies against *P*. *vivax* to *P*. *knowlesi* in the blood stage, and these antibodies can potently inhibit in vitro invasion, highlighting the potential cross-protective immunity in endemic areas.

## Author summary

In recent years, malaria initiatives have increasingly shifted focus from achieving malaria control to achieving malaria elimination. However, the interventions used are leading to drastic changes in the proportions of different *Plasmodium* species causing clinical infection, particularly within Southeast Asia. Little is known about how these different parasite species interact/compete in nature or whether exposure to one species could cause some level of protection against another. We examined cross-reactive antibody responses to key parasite proteins with roles in red blood cell invasion and identified novel cross-species reactivity among the closest of malaria affecting the human population (*P*. *vivax* and *P*. *knowlesi*). This comprehensive analysis provides evidence that cross-reactive immunity could play an important role in areas where species distributions are perturbed by malaria control measures, and future efforts to identify the specific cross-reactive epitopes involved would be invaluable both to our understanding of malaria immunity and vaccine development.

## Introduction

Malaria remains a deadly scourge in humans, with over 200 million cases and close to a half-million deaths reported annually [1]. Protozoan malaria parasites are transmitted by sporozoite inoculation by a mosquito vector into the human [2]. Five *Plasmodium* species infect humans; *Plasmodium falciparum*, *P*. *vivax*, *P*. *ovale*, *P*. *malariae*, and zoonotic malaria parasite *P*. *knowlesi* [2,3]. *P*. *falciparum* is the most virulent species and leads to most severe cases in Africa, while *P*. *vivax* is widely distributed and predominates in regions of Southeast Asia and South America [1]. Recently, a monkey malaria parasite, *P*. *knowlesi*, has been found to be cause a significant number of human infections in Southeast Asian countries [4–7].

In recent years, malaria initiatives have increasingly shifted focus from achieving malaria control to achieving malaria elimination. Interventions are leading to drastic changes in the proportions of different *Plasmodium* species affecting populations. For example, as *P*. *vivax* forms latent hypnozoite stages, infection rates are often slower to decline under control measures that *P*. *falciparum* [8]. In most areas where malaria is endemic, two or more human parasites coexist [9], and mixed infections with three malaria parasite species (*P*. *falciparum*, *P*. *vivax*, and *P*. *knowlesi*) have been identified in southern Myanmar, Thailand and Sabah, Malaysia [10–12]. However, little is known about how the different parasite species interact/compete in nature or whether exposure to one species could cause some level of protection against another. If cross-species immunity is important, the successful control of one species could lead to increases in the frequency or severity of infections from other *Plasmodium* species [9,13]. Previous studies looking at patients concurrently infected with multiple *Plasmodium* species demonstrated clear cross-regulatory patterns, including evidence for density-dependent regulation of parasitaemia and peak parasitaemias for each parasite forming sequential patterns [14]. Density-dependent regulation of parasitemia and specific immune responses targeting particular antigens could explain the prevalence of coinfection patterns in endemic areas [14].

An extreme example of how malaria control can affect species distribution is in Malaysia, where successful control of human malaria has led to a significant decline in *P*. *falciparum* and *P*. *vivax* cases at the same time as zoonotic *P*. *knowlesi* cases have dramatically increased. For example, in Sabah, *P*. *knowlesi* caused less than 5% of malaria cases in 2004, but in 2017, it was responsible for 98% of cases [15,16]. Equally, it is also possible that the removal of *P*. *falciparum* or *P*. *vivax* from populations could have an effect on the spread or pathogenicity of other human parasites such as *P*. *ovale* and *P*. *malariae* which often cause subclinical infections [17].

However, cross-species immunity in malaria has been largely ignored in the last few decades [14]. Previous reports have observed cross-reactivity between *P*. *falciparum* and *P*. *malariae* and between *P*. *falciparum* and *P*. *vivax* due to conserved/homologous plasmodial proteins and the presence of shared B- and T-cells epitopes [18–25]. *P*. *vivax* antigens were recognized by sera from *P*. *vivax* and *P*. *falciparum* clinical patients [20,22]. Naturally acquired antibodies in *P*. *falciparum*-infected patients inhibit the invasion of hepatocytes by rodent malaria sporozoites [18]. Previous reports found that past exposure to *P*. *vivax* or *P*. *malariae* leads to lower morbidity and mortality following *P*. *falciparum* exposure [14,26]. However, little is known about which factors affect the cross-reactivity observed in those previous reports. In addition, the identification of species-transcending inhibitory antibodies could provide a powerful tool for vaccine development, particularly as these antibodies are far more likely to result in strain-transcending effects, which has been a major challenge in malaria vaccine research [27].

*P*. *knowlesi* and *P*. *vivax* are phylogenetically closely related [28], and approximately 89% of *P*. *vivax* genes have orthologues in *P*. *knowlesi* [29]. In silico analysis has demonstrated that there is a high degree of homology among plasmodial proteins, especially those of *P*. *vivax*, *P*. *knowlesi* and *P*. *cynomolgi* [28]; thus, cross-reactivity may play an important role in the prevalence patterns observed in endemic areas of Southeast Asian countries where both human and primate malaria coexist. It also remains interesting to explore the existence of cross-reacting epitopes in blood-stage malaria vaccine candidates including *P*. *knowlesi* [30]. The disease dynamic change has been seen in Malaysia while decreasing human *Plasmodium* species is observed, the increasing of *P*. *knowlesi* infection is inevitable [15]. The possibility of waning cross-protective *P*. *vivax* antibodies may contribute to increased zoonotic *Plasmodium* infection [31]. It is in line with our previous study that *P*. *vivax* antibodies inhibited the *P*. *knowlesi* invasion in vitro [25].

This study presents a systematic analysis of cross-species reactivity of antibodies against *P*. *vivax* blood-stage antigens and their inhibition activities against human erythrocyte invasion by *P*. *knowlesi*. We also observed that antigen-specific IgG antibodies from *P*. *vivax*-infected patients could inhibit erythrocyte invasion in *P*. *knowlesi* parasites. These data indicate that significant cross-species reactive antibodies are generated during malaria infections and that these may play a role in susceptibility or pathogenicity, particularly between closely related species like *P*. *vivax* and *P*. *knowlesi*. It suggests that the potential for a developed vaccine against multiple *Plasmodium* species should be examined in more detail.

## Materials and methods

### Ethics statement

Blood samples were taken from human subjects, age 18–70 years old after written-informed consent was obtained from all subjects. All experimental protocols involving human samples were approved by the Kangwon National University Hospital Ethical Committee (IRB No. 2014-08-008-002), the University of Malaya Medical Ethics Committee (Ref No. 817.18), and the Medical Research Ethics Committee (MREC), Ministry of Health, Malaysia (National Medical Research register ID No. 13079), in accordance with relevant guidelines and regulations. Blood samples were collected from *P*. *knowlesi*-infected patients in EDTA-vacutainer tubes provided by the University of Malaya Medical Center (UMMC), Malaysia during 2010–2013, on the day when the patients were positively diagnosed with malaria. All *P*. *knowlesi* samples used for this study were confirmed by species-specific PCR as described elsewhere [4]. The patients’ serum were collected from symptomatic patient visiting at endemic areas of Malaysia with parasitemia 0.207% in average (ranging from 0.003 to 1.380%) and age 38 years in average (range from 6 to 72). However, we did not obtain information about history of previous malaria infection from patients. *P*. *vivax*-infected patients were recruited from a *P*. *vivax*-endemic area in Gangwon province, the Republic of Korea (ROK) where *P*. *vivax* is major *Plasmodium* species, there is no *P*. *knowlesi* infection in this area [1]. Patients with *P*. *falciparum* infection were excluded in this study. The sera were collected from symptomatic patient visiting at local health centers and clinics in Gangwon Province, ROK with parasitemia ranging from 0.027 to 0.630%, mean 0.121%) and age 18 to 60 years (mean 28). *P*. *vivax* and *P*. *knowlesi* eight patient serum samples were pooled after species-specific PCR and eight health individuals without any *Plasmodium* infection. A total 70 individual *P*. *vivax-*, *P*. *knowlesi*-infected patients serum and health individuals were used for individual immunoscreening after preliminary pooled-serum screening. Patients without malaria were recruited from a population of healthy individuals living in nonendemic areas of the ROK; malaria negativity was confirmed by microscopy and PCR.

### Sequence analysis of amino acid identity

The target antigens were selected from our previous studies that were localized in different apical organelles/ merozoite surface of *P*. *vivax* and were recognized by human patients serum (Table 1, see references). Amino acid sequences of *P*. *vivax* (Sal I and P01 strains) and *P*. *knowlesi* (H strain) were retrieved from PlasmoDB (www.plasmodb.org) [32] and aligned. Clustal W program was used to make a pairwise alignment to determine the percent amino acid identity (Table 1 and S1 Fig).

**Table 1:** Characterization of *P*. *vivax* blood-stage antigen-specific antibodies, colocalization and the degree of similarity of homologous domains.

*P*. *vivax* Sal-I					*P*. *knowlesi* strain H				
Name	PlasmoDB ID (Sal-I)	MW (full length/ functional domain)	Localization	Ref.	PlasmoDB ID	% identity (aa.) of functional domain with two *P*. *vivax* strains		Colocalization	Ref
						Sal-I	P01		
PvMSP1P	PVX_099975	214.6/9.6	Surface	[33]	PKNH_0728800	86.0	87.5	Surface	[34]
PvMSP1	PVX_099980	196.1/9.8	Surface	[35]	PKNH_0728900	82.0	87.4	Surface	[36]
PvMSP10	PVX_114145	52.3/47.0	Surface	[37]	PKNH_1129800	62.4	63.0	Surface	[38]
PvMSP8	PVX_097625	54.7/49.3	Surface	[39]	PKNH_1031500	82.8	84.1	ND	[38]
Pv41	PVX_000995	44.1/41.7	Surface	[40]	PKNH_0303000	83.7	83.7	Surface	[41]
Pv50	PVX_087140	50.4/48.4	Surface	[42]	PKNH_0730000	58.2	57.8	Surface	-
Pv32	PVX_084815	32.6/26.7	Surface	[43]	PKNH_0421000	77.3	76.8	Surface	-
PvMSA180	PVX_094920	182.1/43.0 (N) 57.9 (C)	Surface (N & C)	[44]	PKNH_0814000	61.8 (N) 74.5 (C)	61.6 (N) 74.9 (C)	Surface (N & C)	-
PvGAMA	PVX_088910	82.7/57.5	Microneme	[45]	PKNH_1322900	82.4	82.5	Microneme	-
PvDBP	PVX_110810	119.7/38.5	Microneme	[46]	PKNH_0623500	71.4	70.7	ND	[47]
PvAMA1	PVX_092275	64.5/64.5	Microneme	[42]	PKNH_0931500	85.4	85.0	Microneme	[47]
PvRBP1a	PVX_098585	326.3/34.4	Microneme	[48]	PkNBPXa PKNH_1472300 PkNBPXb PKNH_0700200	22.9 (Xa) 22.3 (Xb)	20.2 (Xa) 24.3 (Xb)	Microneme	-
PvRBP1b	PVX_098582	303.7/32.0	Microneme	[48]	PkNBPXa PKNH_1472300 PkNBPXb PKNH_0700200	26.9 (Xa) 31.3 (Xb)	16.7 (Xa) 14.6 (Xb)	Microneme	-
Pv12	PVX_113775	41.1/36.0	Rhoptry neck	[49]	PKNH_1137300	74.9	75.2	Rhoptry	NA
PvRON2	PVX_117880	244.6/68.9	Rhoptry neck	[42]	PKNH_1230100	71.0	78.0	ND*	NA
PvRAMA	PVX_087885	81.3/27.9	Rhoptry body	[50]	PKNH_0105800	74.6	75.6	ND**	NA
PvRhopH2	PVX_099930	160.9/42.6	Rhoptry body	[51]	PKNH_0727900	73.3	71.2	Rhoptry	NA
PvETRAMP 11.2	PVX_003565	11.9/9.5	PVM	[52]	PKNH_0418600	74.7	80.2	ND	NA
PvEXP1	PVX_091700	15.0/12.7	PVM	[52]	PKNH_0919300	77.5	79.6	ND	NA

Identity of amino acid sequences was obtained based on the alignment of the target region shown in S1 Fig and colocalization on *P*. *knowlesi* parasites was determined by the *r*^*2*^ value (>70%) in S1 Table.

MW, predicted molecular weight; FL, full length; NA, not assigned; ND: not defined; PVM, parasitophorous vacuole membrane; N, N-terminal; and C, C-terminal.

*, probably on the rhoptry neck

**, not in the rhoptry bulb.

### In vitro culture of blood-stage *P*. *knowlesi* parasites

The human-adapted *P*. *knowlesi* A1-H.1 strain was maintained with fresh human erythrocytes in RPMI 1640-based medium (Invitrogen/Life Technologies, Grand Island, NY) as described previously [53].

### Recombinant protein expression

*P*. *vivax* recombinant proteins were generated by wheat germ cell-free or *E*. *coli* expression systems in our previous studies [25,42,44,50]. DNA fragments encoding PvRBP1a-34, Pv41, or PvRhopH2 were amplified from a Korean vivax isolate with primers PvRBP1a-34_F (ggtcgcggatccgaattcATGAACGAACTAGGTATAGACATT) and PvRBP1a-34_R (ggtggtggtgctcgagTTCAAACTCTATCTTCAGTTC); Pv41_F (ggtcgcggatccgaattcATGGAACACATCTGCGATTTTAC) and Pv41_R (ggtggtggtgctcgagCTCCTGGAAGGACTTGGCA); and PvRhopH2_F (ggtcgcggatccgaattcATGGAGCTGAGCCACAGC) and PvRhopH2_R (ggtggtggtgctcgagCTTCTCCACATCCTCGTGGT), respectively. Small letters indicate the plasmid-derived sequence and underlined letters indicate enzyme restriction sites, EcoRI and XhoI, respectively. High fidelity KOD-plus kit (Toyobo Co., Osaka, Japan) was used with an initial denaturation step at 94°C for 2 min, followed by 35 cycles of 94°C for 15 sec, 60°C for 30 sec, and 58°C for 1.5 min and a final extension step at 68°C for 10 min. The amplicons were purified using a gel extraction kit and ligated into the pET28a(+) expression vector (Novagen, Madison, WI) for PvRBP1a-34 or the pET23a(+) expression vector for Pv41 and PvRhopH2 with a His-tag. Correctly ligated plasmids were transformed into *E*. *coli* BL21(DE3) pLysS (Life Technologies) for recombinant protein expression. Recombinant protein expression was induced with 0.1 mM isopropyl-β-D-thiogalactopyranoside (IPTG; Sigma-Aldrich, St. Louis, MO). All recombinant proteins were solubilized, purified, and refolded as described elsewhere [47,54].

Meanwhile, *P*. *knowlesi* recombinant proteins were expressed for this study using wheat germ cell-free system for immunoscreening using specific primers (S2 Table), the procedures were described elsewhere[47]. Recombinant PkDBPα protein expression was described elsewhere [47]. The DNA fragment encoding PkRhopH2 was amplified from *P*. *knowlesi* A1-H.1 genomic DNA with primer pair PkRhopH2_F (ggtcgcggatccgaattcATGGAGTTAGGCCATACCGTG) and PkRhopH2_R (ggtggtggtgctcgagCTTCTCGATGTCTTCGTAGTCCA). Lowercase letters indicate the plasmid-derived sequence and underlined letters indicate the enzyme restriction sites for EcoRI and XhoI, respectively. A high fidelity KOD-plus kit was used with an initial denaturation step at 94°C for 2 min, followed by 35 cycles of 94°C for 15 sec, 60°C for 30 sec, and 58°C for 1.5 min and a final extension step at 68°C for 10 min. The amplicons were purified using a gel extraction kit and ligated into the pET28a(+) expression vector with a His-tag. Correctly ligated plasmids were transformed into *E*. *coli* BL21(DE3). The protein expression and purification was performed as described above. *P*. *knowlesi* MSP1-19 protein was cloned into pGEX-4T-2 vector (GE Healthcare Life Sciences, Uppsala, Sweden) by In-Fusion cloning according to manufacturer’s manual, and the cloned pDNAs were transformed into BL21(DE3) competent cells for recombinant protein expression. The proteins were purified with Glutathione Sepharose 4B (GE Healthcare Life Sciences) and used for mice antibody production.

### SDS-PAGE and western blot analysis

Parasite lysates were prepared from *P*. *knowlesi* schizont as described previously [55]. Recombinant proteins were separated by 13% SDS-PAGE under reducing or non-reducing conditions and stained with 0.25% Coomassie brilliant blue R-250 (Sigma-Aldrich) and used for western-blotting analysis [25]. The membrane-transferred proteins were reacted with primary rabbit polyclonal serum (1:50) or an anti-His monoclonal antibody (1:2,000, Hilden, Hamburg, Germany) and then reacted with secondary IRDye-labeled goat anti-rabbit or goat anti-mouse antibodies (1:10,000) (LI-COR Bioscience, Lincoln, NE). An Odyssey infrared imaging system (LI-COR Bioscience) and Odyssey software (LI-COR Bioscience) were used to visualize the bands.

### Animal antibody production and IgG purification

All *P*. *vivax* blood-stage antigen-specific antibodies were obtained during our previous studies (Table 1, S3 Table). Total IgG was purified from 1 mL of rabbit serum by using a protein G HP column according to the manufacturer's protocol (GE Healthcare Life Sciences) as described elsewhere [47]. All animal experimental protocols were approved by the Institutional Ethics Committee and followed the Ethical Guidelines for Animal Experiments of Kangwon National University (KIACUC-16-0158).

### Antigen-specific IgG antibody purification from patient serum samples

The serum samples used in this study were pooled from eight *P*. *vivax*-infected patients from the ROK with a high response to the particular antigen of interest, which was screened by immunoscreening with a protein microarray. The control comprised pooled serum samples from eight healthy individuals from a nonendemic area of the ROK. Total IgG antibodies were purified from the pooled serum samples by using protein G columns according to the manufacturer’s protocol (GE Healthcare Life Sciences). The isolated IgG antibodies were dialyzed against RPMI 1640 medium and concentrated using centrifugal devices (Merck Millipore, Darmstadt, Germany) with a 30-kDa cut-off value to a concentration of 10 to 20 mg/mL. PvRBP1a, Pv41, and PvRhopH2 recombinant proteins (2–3 mg each) were immobilized on cyanogen bromide (CNBr)-activated Sepharose 4 fast flow beads (GE Healthcare Life Sciences) according to the manufacturer’s instructions. The total isolated IgG antibodies (0.5 mL) was loaded to the column filled with antigen coupled beads and eluted using an elution buffer (0.1 M glycine, pH 2.7). The eluted antigen-specific IgGs were immediately neutralized with a Tris buffer (pH 9.0) and dialyzed against incomplete RPMI 1640 medium to the desired concentration.

### Immunofluorescence assay (IFA)

An IFA was performed as described previously [47]. The slides were dual probed with a panel of rabbit polyclonal serum against *P*. *vivax* antigens (1:50) and mice serum against *P*. *knowlesi* antigens as localization markers (PkMSP1-19 for merozoite surface, PkDBPα-II for microneme, and PkRhopH2 for rhoptry; 1:50). Alexa Fluor 488 goat anti-mouse IgG (H+L) and Alexa Fluor 568 goat-anti rabbit IgG (H+L) were used as secondary antibodies. Nuclei were stained with 4’,6-diamidino-2-phenylindole (DAPI, Invitrogen). Red and green pixel intensities were analyzed by ImageJ. The scatter plots of individual red and green pixel intensities were then compared and coefficient of determination (*r*-squared) was obtained. The larger *r*-squared (as close to 100%) represents the scatter value around the regression line suggesting the variation of red and green pixel intensities around its mean. Colocalization was determined as spatial overlap between red (control) and green pixel intensities as showed in larger *r*-squared value.

### Protein microarray

The protein microarray protocol was described elsewhere [25]. Serum was pooled from eight *P*. *vivax*- and *P*. *knowlesi-*infected patients, healthy donors and/or individually used without pooling to evaluate cross-immunoreactivity. The pooled or individual *P*. *vivax*- or *P*. *knowlesi*-infected patient serum or healthy serum were diluted as 1:25. The cut-off value was equal to the mean fluorescence intensity (MFI) plus two standard deviations (SDs) of the negative samples. Normalized MFI values were calculated from the MFI/cut-off values. The normalized MFI for the pooled patient serum was subtracted from the corresponding normalized MFI for the pooled healthy serum.

### Growth inhibition assay

The standardized protocol for the invasion inhibition assay has been described elsewhere [47]. Briefly, 2 mg/mL purified rabbit IgG antibodies, and a concentration gradient of antigen-specific IgG antibodies from patients (0.1, 0.2, and 0.5 mg/mL) was added to a 96-well culture plate containing *P*. *knowlesi* schizonts with 1.5% initial parasitemia and 2% hematocrit from fresh human erythrocytes. An anti-Fy6 monoclonal antibody (25 μg/mL), which recognizes the 2C3 epitope in the DARC N-terminal region located on the red blood cell surface membrane, was obtained as previously described [56]. This antibody was a kind gift from Dr. Olivier Bertrand and Dr. Yves Colin (Institut National de la Transfusion Sanguine, Paris, France). After 10 h of post-invasion for *P*. *knowlesi*, the parasites were stained with SYBR Green I (Sigma-Aldrich) and analyzed with an Accuri C6 flow cytometer (Accuri cytometer Inc., Ann Arbor, MI). Two independent experiments were performed in duplicate; three independent experiments in duplicate were not possible due to limited antibody amount.

### Data analysis

All calculations and data analysis were performed with GraphPad PRISM 5 (GraphPad Software, Inc., San Diego, CA). The Mann-Whitney test was used to assess differences between means, and one-way ANOVA with Dunnett’s test was used to compare the means from more than two groups with control anti-HisGST; 95% confidence intervals (CIs), and *p* < 0.05 was considered significant. The 50% inhibitory concentration (IC_50_) was plotted against log-transformed antibody concentrations, and curve fitting by nonlinear regression with Excel software was used to identify 50% parasite growth inhibition. Hierarchical clustering was used to calculate the profile of individual seropositivity using TIGR multiarray experiment viewer (MeV) software [57]. The clustering analysis was performed using average linkage clustering with Euclidean distance as a similarity metric.

## Results

### *P*. *vivax* blood-stage antigens are similar to those of *P*. *knowlesi*

Twenty rabbit antibodies previously generated against 19 *P*. *vivax* blood-stage vaccine candidates were evaluated for their cross-species reactivity against *P*. *knowlesi* (S1 Fig). Of those, most of the *P*. *vivax* antigens were highly expressed, and previous studies localized 8 to the merozoite surface, 5 microneme molecules, 4 rhoptry molecules, and 2 dense granule molecules (Table 1). The sequence alignment with orthologs of *P*. *knowlesi* (*n* = 18) revealed a high degree of similarity, on average 75.2% for *P*. *vivax* Sal-I and 72.4% for P01 strain., in the regions selected for antibody production (Table 1).

### *P*. *vivax* blood-stage antigen-specific antibodies bind to *P*. *knowlesi* antigens

**Fig 1:**
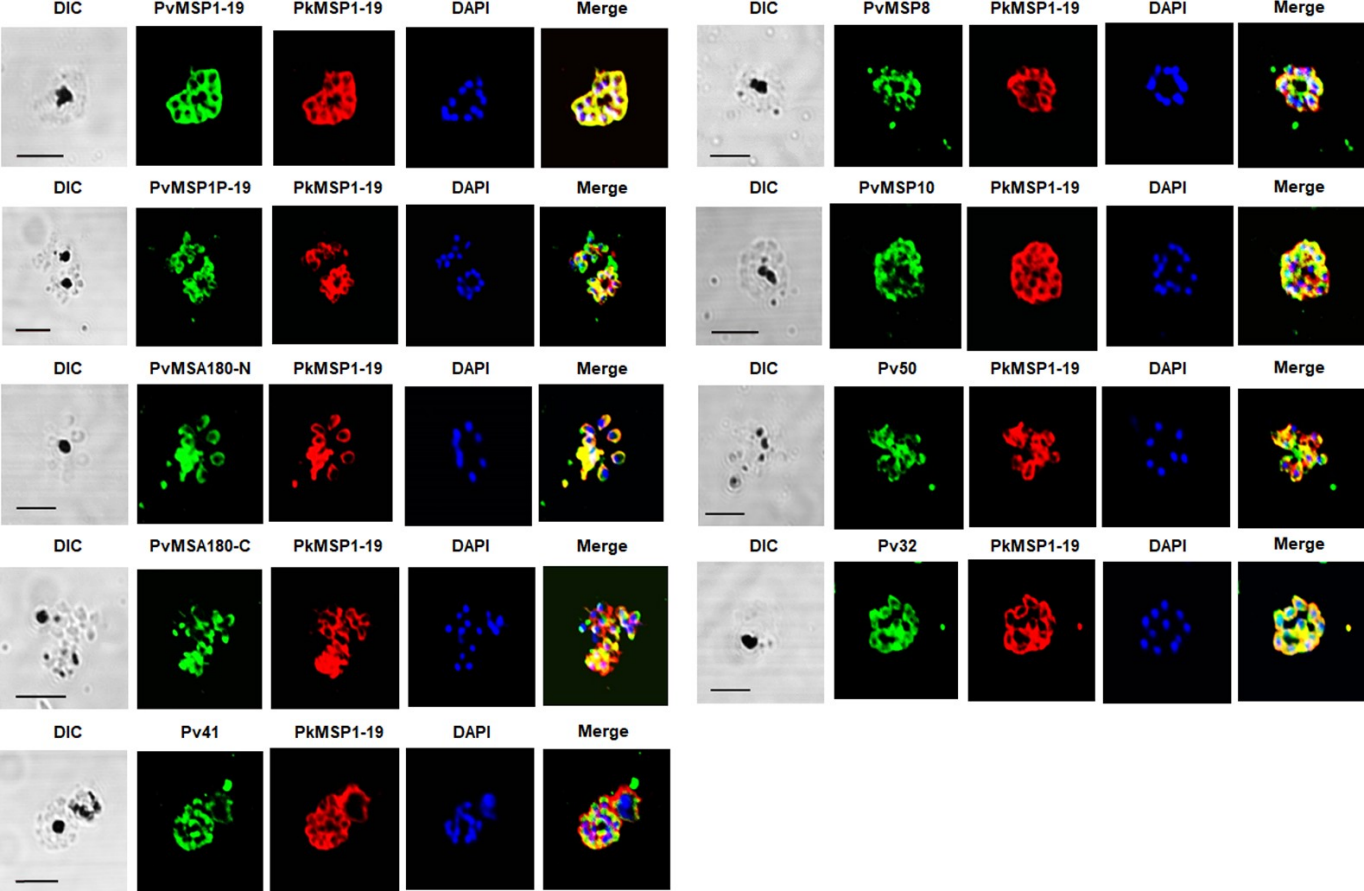
Cross-species reactivity of antibodies against *P*. *vivax* merozoite surface proteins to *P*. *knowlesi* parasites by immunofluorescence assay. Localization of a panel of antibodies for *P*. *vivax* surface proteins (green) in *P*. *knowlesi* co-stained with anti-PkMSP1-19 (red) as a surface marker. DIC, differential interference contrast; DAPI, 4’,6-diaminidino-2-phenylindole (blue). All parasites shown are segmented schizonts (24–28 hours post-invasion). Bars indicate 5 μm.

**Fig 2:**
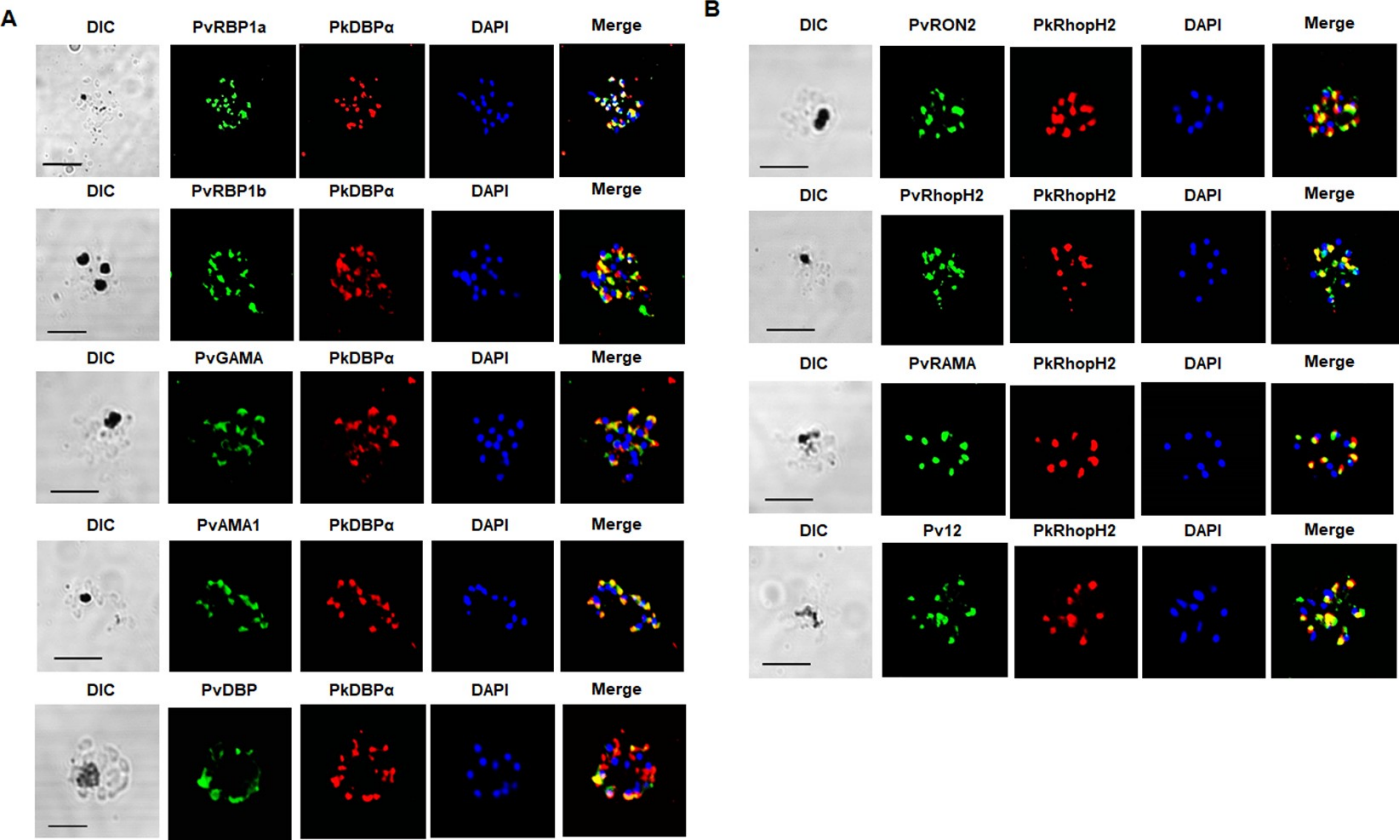
Cross-species reactivity of antibodies against *P*. *vivax* apical organelles proteins to *P*. *knowlesi* parasites by immunofluorescence assay. (A) Localization of a panel of antibodies for *P*. *vivax* micronemal proteins (green) in *P*. *knowlesi* co-stained with anti-PkDBPα (red) as a microneme marker and DAPI as nuclear marker (blue). (B) Localization of a panel of antibodies for *P*. *vivax* rhoptry proteins (green) in *P*. *knowlesi* co-stained with anti-PkRhopH2 (red) as a rhoptry marker and DAPI as nuclear marker (blue). All parasites shown are segmented schizonts (24–28 hours post-invasion). Bars indicate 5 μm.

**Fig 3:**
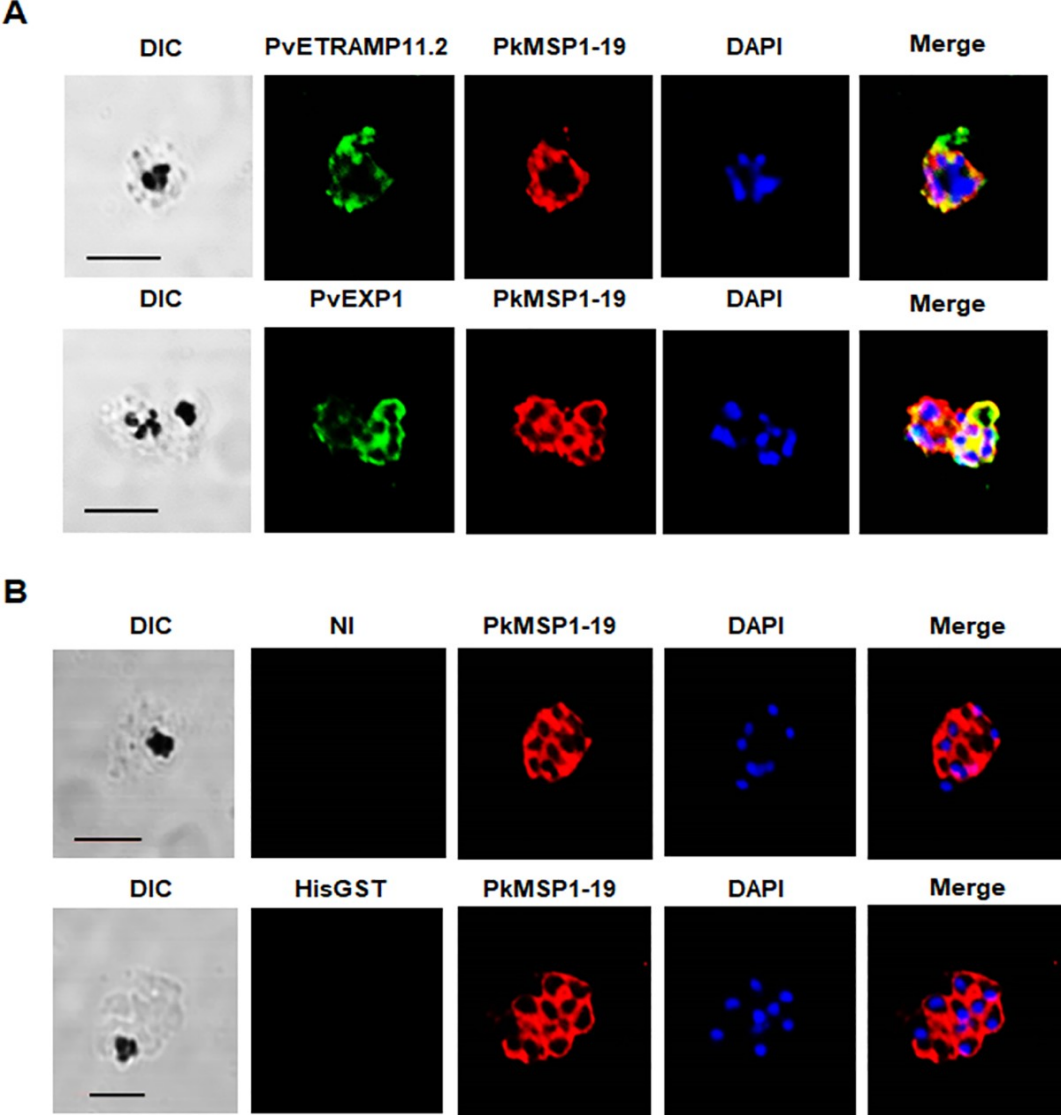
Cross-species reactivity of antibodies against *P*. *vivax* parasitophorous vacuole membrane molecules and negative control antibodies to *P*. *knowlesi* parasites by immunofluorescence assay. (A) Localization of antibodies for *P*. *vivax* parasitophorous vacuole proteins (green) in *P*. *knowlesi* co-stained with anti-PkMSP1-19 as a merozoite surface marker. (B) Localization of PBS-immunized (NI) and HisGST antibodies to *P*. *knowlesi*. All parasites shown are segmented schizonts (24–28 hours post-invasion). Bars indicate 5 μm.

Immunofluorescence assay (IFA) revealed that these antibodies recognized mature schizont- or merozoite stage parasites of *P*. *knowlesi* (Figs 1–3), whereas antibodies from a PBS-immunized rabbit (NI) and antibody raised against recombinant HisGST protein had no specific reactivity to *P*. *knowlesi* parasites (Fig 3B). For the analysis of cross-reactivity to the surface antigen of *P*. *vivax* merozoites, anti-PvMSP1-19, anti-PvMSP1P-19, anti-PvMSA180-N, anti-PvMSA180-C, anti-Pv41, anti-PvMSP8, anti-PvMSP10, anti-Pv50, and anti-Pv32 antibodies were used. Most of the surface antigens in *P*. *knowlesi* parasites were highly cross-reactive with the 9 *P*. *vivax* antibodies and a positive control anti-PkMSP1-19 antibody (Fig 1). The anti-PvMSP1-19 and PvMSA180-N antibody showed the strongest recognition of the surface of the *P*. *knowlesi* merozoite, as shown by full overlap (*r*^2^ > 95%) with the anti-PkMSP1-19 signals (Fig 1, S1 Table).

Most signals with antibodies against *P*. *vivax* microneme proteins, PvRBP1a, PvRBP1b, PvGAMA, PvAMA1, and PvDBP, were colocalized with signals with anti-micronemal PkDBPα antibody in *P*. *knowlesi* parasites (Fig 2A). Interestingly, antibodies against PvRBP1a and PvRBP1b, for which the syntenic orthologs are pseudogenes in *P*. *knowlesi*, showed signals for these parasites (Fig 2A).

Antibodies against *P*. *vivax* rhoptry body antigens (PvRhopH2) and rhoptry neck antigens (PvRON2) cross-reacted with *P*. *knowlesi* showing complete (PvRhopH2) or partial (others) colocalization signals for rhoptry body protein PkRhopH2 in *P*. *knowlesi* parasites, respectively (Fig 2B, S1 Table). Antibodies against PvETRAMP11.2 and PvEXP1, which localize to the parasitophorous vacuole/dense granules, partially overlapped with the MSP1-19 (surface) consistent with a parasitophorous vacuole localization (Fig 3A). The western blot analysis using parasite lysates showed that the *P*. *vivax* antibodies also recognized bands at expected full-length sizes or probably the processing proteins (S2 Fig). PvMSP1-19 and PvRhopH2 antibodies were surprisingly recognized the full length of MSP1 protein in *P*. *knowlesi* lysate. However, non- specific recognition was seen in PvETRAMP11.2 which is in line that the PvETRAMP11.2 antibody did not cross-react with *P*. *knowlesi* parasite organelle protein, but did recognize the parasite membrane.

### Antibodies raised against *P*. *vivax* blood-stage antigens inhibited the erythrocyte invasion by *P*. *knowlesi*

**Fig 4:**
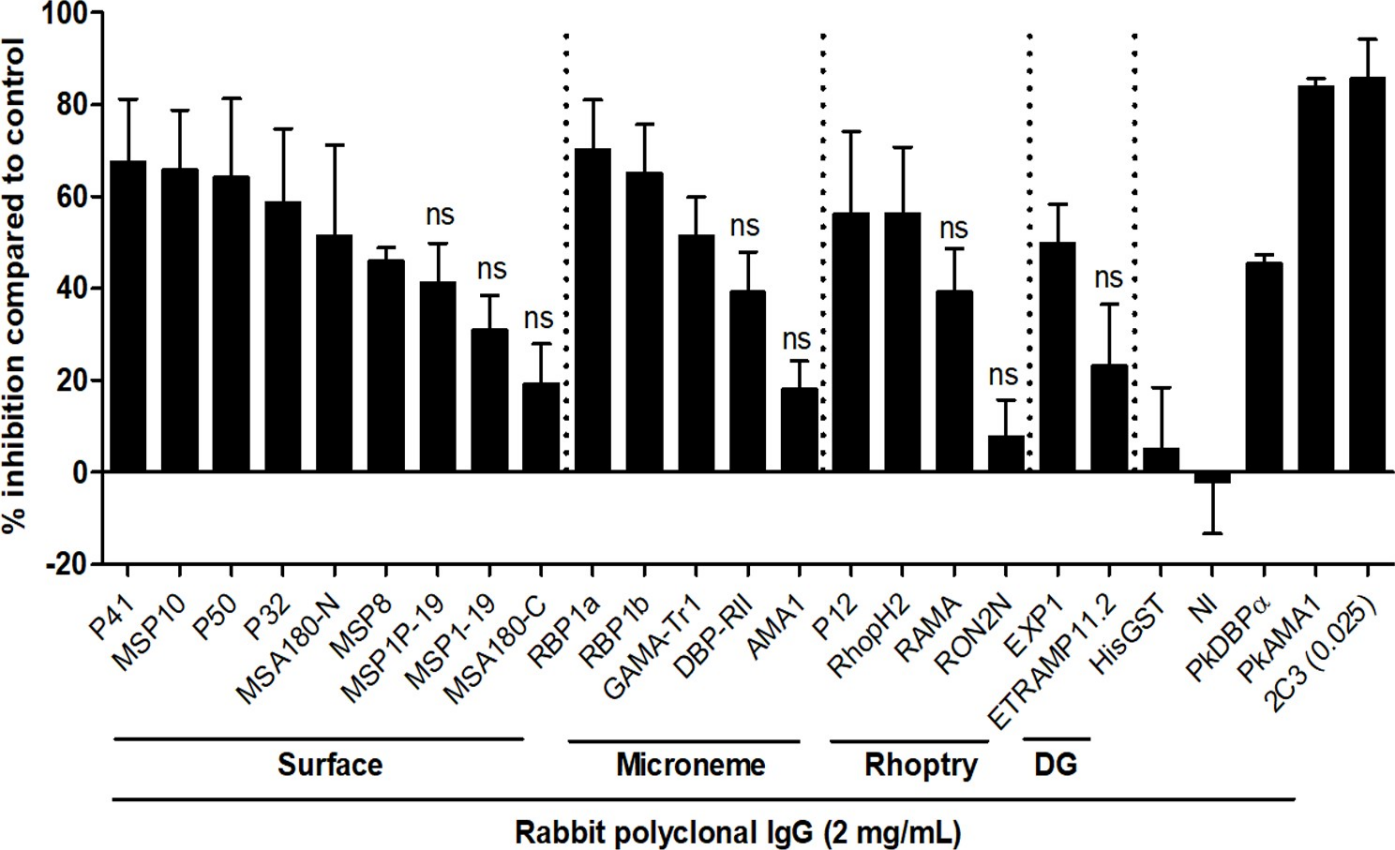
Cross-species activity of antibodies against *P*. *vivax* antigens to inhibit erythrocyte invasion by *P*. *knowlesi* parasites. Graph showing inhibition activity (%) of antibodies against *P*. *vivax* antigens to erythrocyte invasion by *P*. *knowlesi* A1-H.1 (2 mg/mL rabbit IgG). PkDBPα, PkAMA1 rabbit polyclonal IgG, and 2C3 monoclonal antibody served as control. NI, PBS-immunized rabbit IgG; DG, dense granules; 2C3, Anti-Fy6 monoclonal antibody (25 μg/mL). Graphs show the mean and error bars denote ±1 SD of duplicate test wells in two independent experiments by using one-way ANOVA with Dunnett’s multiple comparison test of means of antibody inhibition rate with mean of control anti-HisGST. ns, no significant difference, *p*>0.05.

We next investigated if antibodies raised against *P*. *vivax* antigens block human erythrocyte invasion by *P*. *knowlesi* in vitro. We found that the invasion of *P*. *knowlesi* was inhibited by all antibodies except RON2 when 2 mg/mL of *P*. *vivax* rabbit polyclonal IgG were administered (Fig 4). The inhibition levels varied between the different antigens, but several were higher than 60% (P41, MSP10, P50, RBP1a, RBP1b). Mild inhibition with dose-dependen manner was observed with PvMSP1-19 and PvAMA1 antibodies (Fig 4, S3 Fig). The negative control antibodies, anti-HisGST and PBS-immunized rabbit IgG (anti-NI) showed little to no inhibition, respectively. Administration of 2C3 monoclonal antibody, which targets Duffy antigens on the erythrocyte, resulted in a high degree of inhibition (92.8 ± 1.6%) (Fig 4). Control antibodies, anti-PkDBPα, and anti-PkAMA1 antibodies showed inhibition activity with dose-dependent manner as described in our previous study [47] (S3 Fig).

To determine whether these antibodies demonstrate additive erythrocyte invasion inhibition, a combination of antibodies against three different antigens localized on the surface (Pv41), microneme (PvRBP1a), and rhoptry (PvRhopH2) were combined in a total concentration of 2 mg/mL and evaluated. We found that a combination of these antibodies significantly increased the invasion inhibition efficacy than single antibodies (S4 Fig).

### Human antibodies against *P*. *vivax* blood-stage antigens inhibit erythrocyte invasion by *P*. *knowlesi*

**Fig 5:**
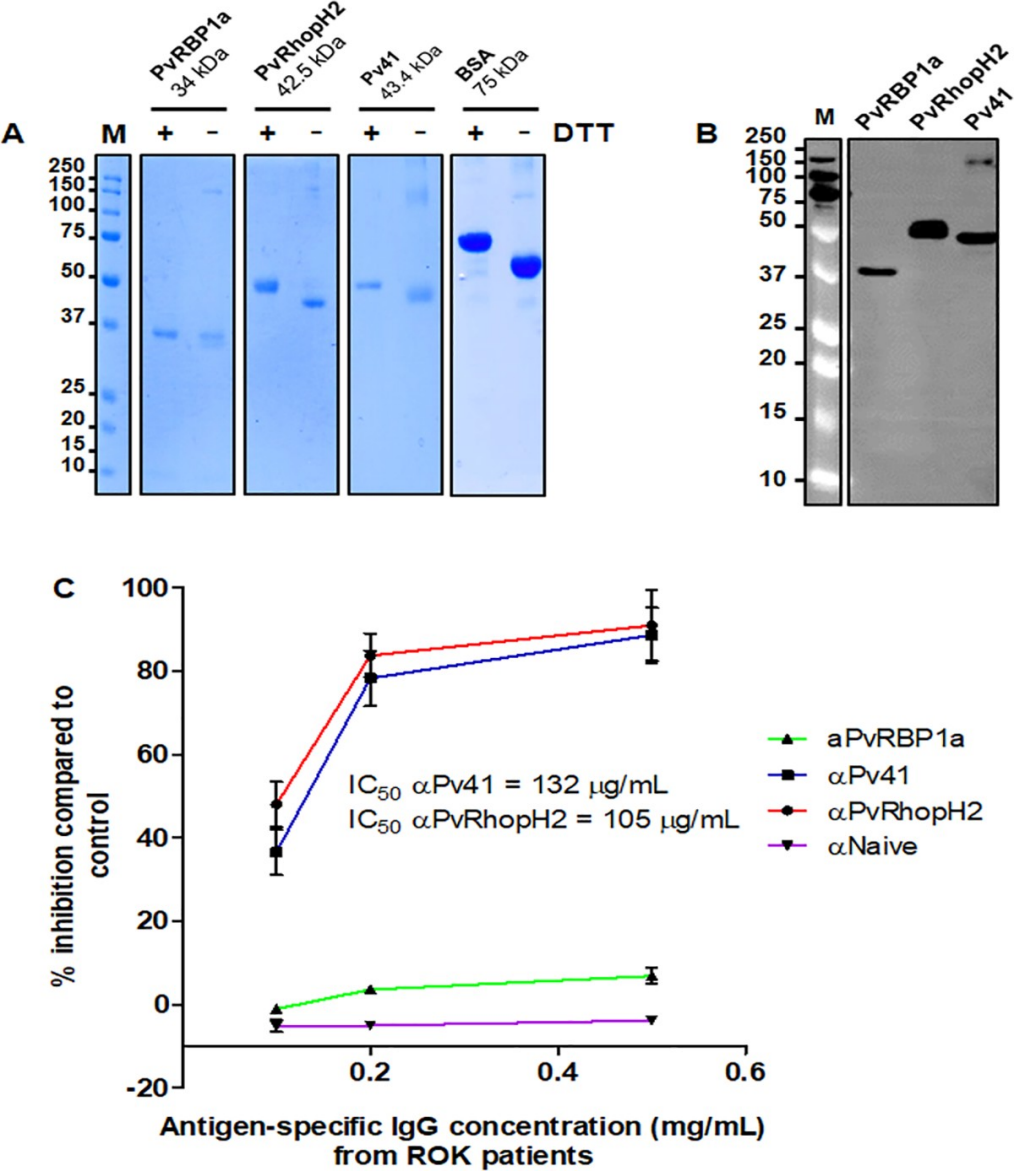
Growth inhibition activity of antigen-specific IgG from *P*. *vivax*-infected patients to *P*. *knowlesi* parasites. (A) Different migration on SDS-PAGE of reduced and non-reduced recombinant proteins immobilized on agarose beads. Proteins were successfully refolded as shown with Coomassie Brilliant Blue in different migration patterns with and without DTT treatment. BSA was served as control. Proteins were then used for immobilization with CNBr-bead for antigen-specific antibody purification from *P*. *vivax*-infected patients serum. (B) Western blot analysis of recombinant PvRBP1a, PvRhopH2, and Pv41 proteins with anti-His-tag antibody. (C) Growth inhibition activity of IgGs specific to *P*. *vivax* antigens to *P*. *knowlesi*. Different concentration of antigen-specific antibodies from human(Pv41, PvRhopH2, PvRBP1a) ranging from 0.1, 0.2 and 0.5 mg/mL were used. IC50 of Pv41 antigen-specific human antibodies was higher than PvRhopH2 antigen-specific human antibodies. αNaive indicates IgG purified healthy individual who have never experienced malaria infection.

To confirm the cross-species inhibition activity of antibodies against *P*. *vivax* antigens, we examined whether human antibodies against the *P*. *vivax* antigens in the serum of patients naturally exposed to *P*. *vivax* infection could inhibit *P*. *knowlesi* erythrocyte invasion. Three *P*. *vivax* antigens, PvRBP1a, Pv41, and PvRhopH2 (Fig 5A and 5B) were successfully produced and used for immobilizing on CNBr beads to purify antigen-specific IgGs from the serum of *P*. *vivax*-infected patients. These antigens were selected because each of them exhibited the highest inhibitory activities within the same localization group (merozoite surface, microneme, or rhoptry). The folded and unfolded PvRBP1a was then reacted with *P*. *vivax-*infected patients serum, and showed reduction of positive reactivity to unfolded protein as compared to folded PvRBP1a. It suggested that the protein was also successfully refolded (S5 Fig). The patients living in vivax malaria endemic area in the Republic of Korea, would have had no previous exposure to *P*. *knowlesi* and were used to purify those three antigens specific human IgG. Anti-Pv41- and anti-PvRhopH2-specific IgGs showed a concentration-dependent invasion inhibition activity to *P*. *knowlesi*, whereas anti-PvRBP1a-specific IgG showed a very little inhibition activity to *P*. *knowlesi* compared to naïve human IgG (Fig 5C).

### Serum from knowlesi- or vivax-malaria patients recognized a panel of vivax- and knowlesi recombinant proteins

**Fig 6:**
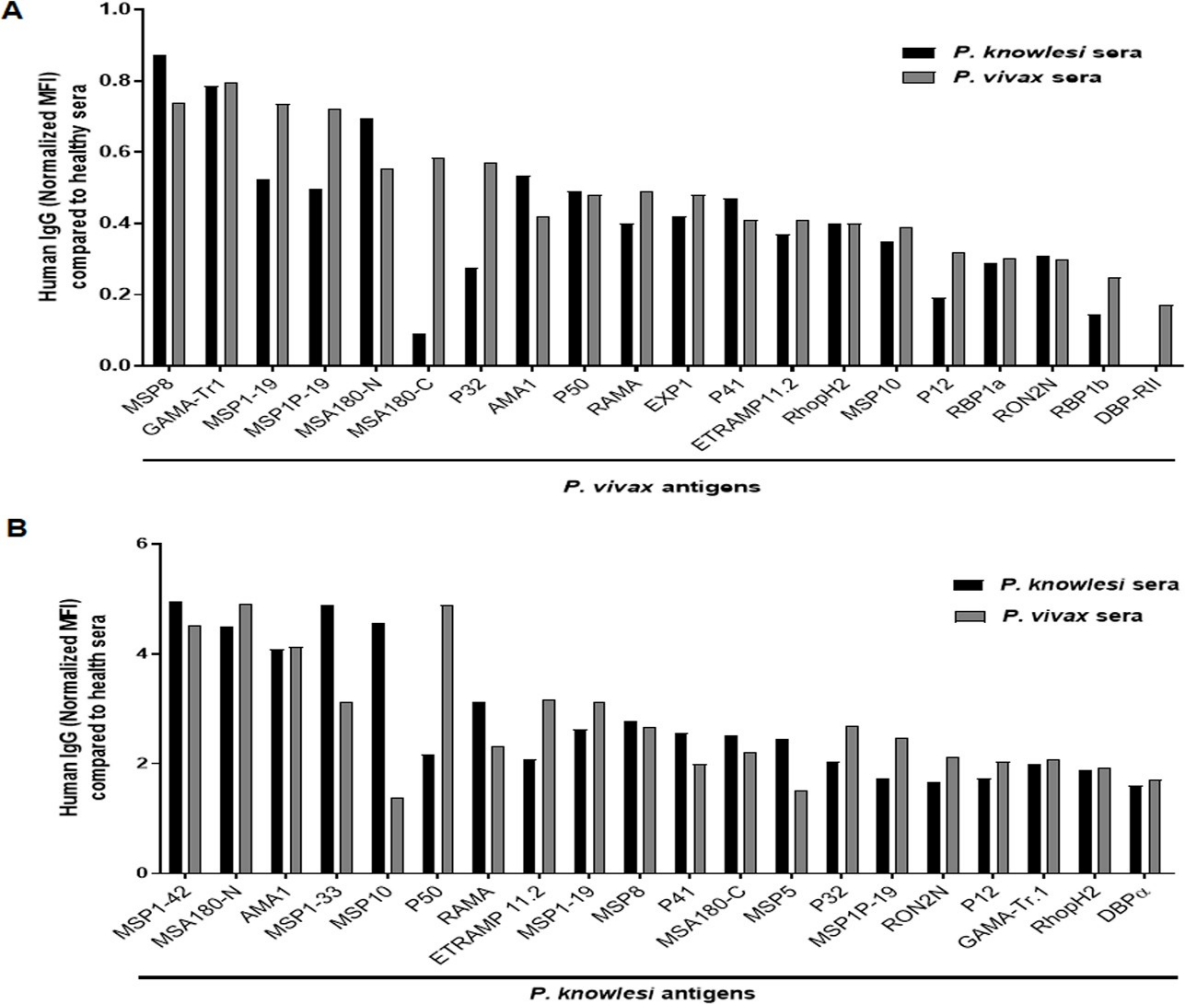
Cross-species reactivity of sera from *P*. *knowlesi*- and *P*. *vivax*-malaria clinical patients against *P*. *vivax* and/or *P*. *knowlesi* proteins. (A) IgG responses of pooled *P*. *vivax*-infected patient serum from the Republic of Korea (ROK) and knowlesi-infected patient serum (Malaysia) with *P*. *vivax* recombinant proteins after subtraction with pooled healthy serum tier in 1:25 dilution. (B) IgG responses of pooled *P*. *vivax*-infected patient serum from the Republic of Korea (ROK) and *P*. *knowlesi*-infected patient serum (Malaysia) with *P*. *vivax* recombinant proteins.

To determine the cross-reactivity in malaria patients living in an endemic area, cross- immunoreactivity of antibodies from *P. knowlesi*- or *P*. *vivax*-infected patients was assessed using a protein microarray of vivax and knowlesi-recombinant proteins. Serum from vivax- and knowlesi-infected malaria patients recognized recombinant *P*. *vivax* and/or *P*. *knowlesi* proteins at different levels of reactivity (Fig 6). PvMSP8 and PvMSA180-N recombinant antigens are found to be more reactive to pooled *P*. *knowlesi-*infected serum (Fig 6A), while Pk50 was very reactive to pooled *P*. *vivax-*infected serum (Fig 6B).

**Fig 7:**
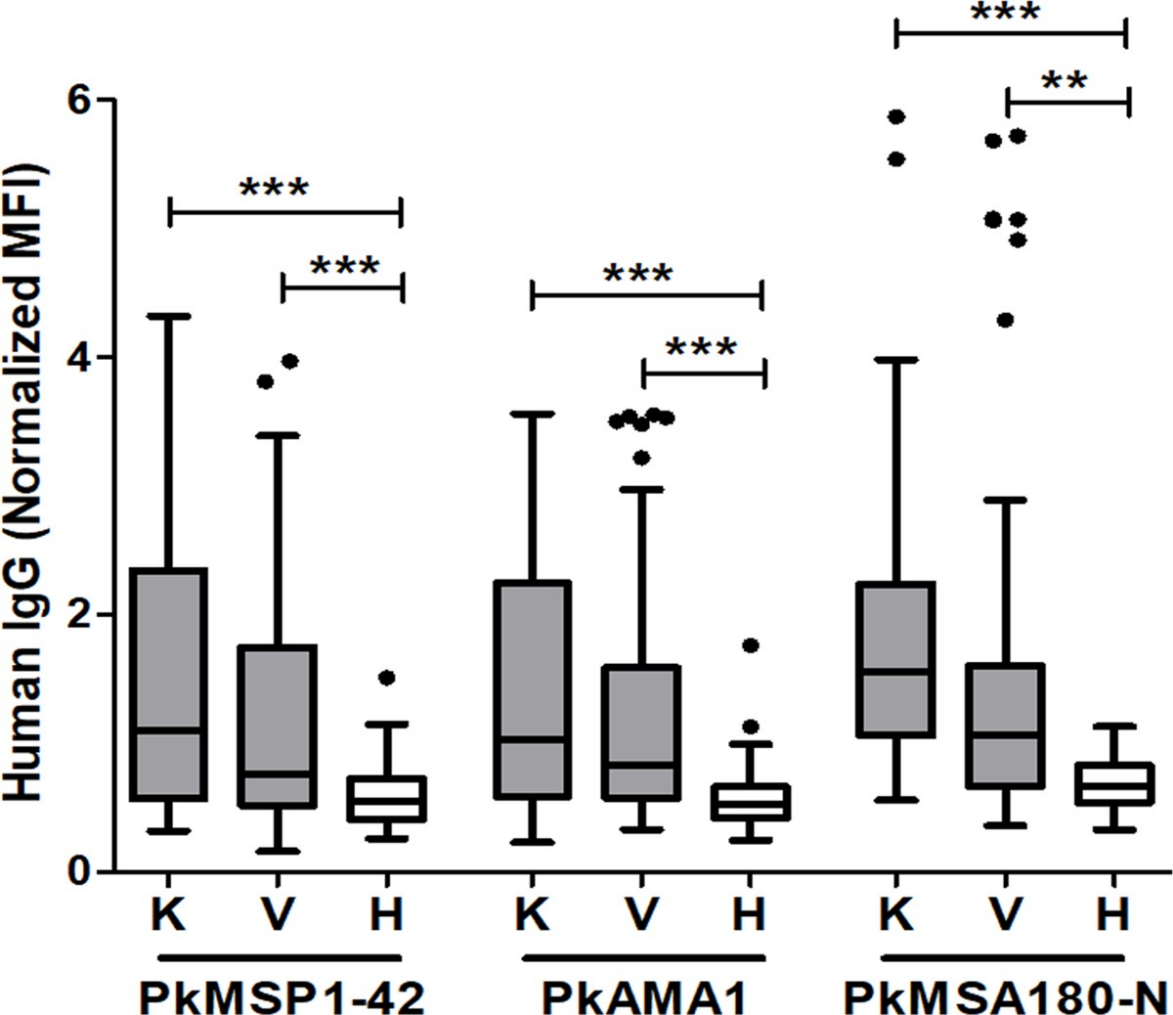
*P*. *knowlesi* blood-stage cross-reactivity with individual patient serum. The human IgG response of PkMSP1-42, PkMSA180-N, and PkAMA1. Individual with outlier reactivity was indicated in black dot. The prevalence of antibody response was compared to the patients (K, knowlesi; V, vivax) and healthy (H) using the Mann-Whitney test. *** = *p*<0.001.

To further investigate the individual immunoprofiling for cross-reactivity, three *P*. *knowlesi* recombinant proteins were used. Three most reactive antigens were selected from immunoscreening results using pooled serum (Fig 6B, S6 Fig). The mean average of seropositive rate of these three antigens was higher to *P*. *knowlesi*-infected patient serum (61.9%) than *P*. *vivax*-infected patient serum (43.3%) (Fig 7, Table 2). Of those, the PkMSA180-N is the most reactive to *P*. *knowlesi*- and *P*. *vivax*-infected patient serum; 77.1 and 51.4%, respectively (Table 2). In total, 37.1% (26/70) of *P*. *knowlesi*-infected patients showed seropositive to all three *P*. *knowlesi* antigens and 27.1% (19/70) of *P*. *vivax*-infected patients showed seropositive to all three *P*. *knowlesi* antigens (S7 Fig). This data indicates that these three proteins are highly immunogenic and posses shared/common epitopes in the two species.

**Table 2:** Seropositivity of IgG responses to top 3 antigens in *P*. *knowlesi* and *P*. *vivax* malaria patients and healthy individuals.

Antigens (*P*. *knowlesi*)	Sample (*n*)	No. of samples		Sensitivity (%)^a^/	95% CI (%)^c^	MFI^d^	*P* value^e^
		Positive	Negative	Specificity (%)^b^			
MSP1-42	*P*. *knowlesi* patient (70)	39	31	55.7^**a**^/	44.1–66.8	1.59	*P* <0.0001
	*P*. *vivax* patient (70)	29	41	41.4^**a**^/	30.6–53.1	1.24	*P* = 0.0008
	Healthy (30)	1	29	96.7^**b**^	83.3–99.4	0.59	
AMA1	*P*. *knowlesi* patient (70)	37	33	52.9^**a**^/	40.0−62.8	1.82	*P* <0.0001
	*P*. *vivax* patient (70)	26	44	41.4^**a**^/	26.8–48.9	1.49	*P* <0.0001
	Healthy (30)	1	29	96.7^**b**^	83.3–99.4	0.68	
MSA180-N	*P*. *knowlesi* patient (70)	54	16	77.1^**a**^/	66.1–85.4	1.49	*P*<0.0001
	*P*. *vivax* patient (70)	36	34	51.4^**a**^/	40.0–62.8	1.27	*P* = 0.0016
	Healthy (30)	1	29	96.7^**b**^	83.3–99.4	0.58	

^**a**^ Sensitivity/ seropositivity rate: percentage of positive in malaria patient samples.

^b^ Specificity/ seronegativity rate: percentage of negative in healthy samples.

^c^ Confidence intervals.

^d^ MFI, mean fluorescence intensity was divided by the cutoff value + 2 standard deviations above the MFI of healthy samples.

^e^ *P* value, the difference in the total IgG level for each antigen between knowlesi or vivax malaria patients and healthy individuals were calculated with the Mann whitney *U*-test. *P* value of <0.05 was considered statistically significant.

## Discussion

Mixed infections are frequently discussed in the context of parasite-parasite interactions among different *Plasmodium* spp. and a high degree of amino acid sequence similarity and shared common epitopes for some parasites antigens suggests a potential role for cross-species immunity [21]. A proof-of-concept study in our laboratory reported that cross-species reactivity between *P*. *vivax* and *P*. *knowlesi* was observed for an apical asparagine-rich protein [25]. In the current study, we characterized antibodies against 20 recombinant proteins derived from 19 *P*. *vivax* blood-stage vaccine candidates for their cross-reactivity to *P*. *knowlesi*. Genome analysis of *P*. *knowlesi* revealed approximately 80% of genes that are orthologous to *P*. *vivax*, *P*. *falciparum*. Among the 19 blood-stage antigens, we selected domains for expression based on their importance for immune response or host cell receptor recognition that we have found in our previous studies (Table 1). Whilst the majority of these had direct orthologues in *P*. *knowlesi*, some did not, including PvRBP1a and PvRBPb which are found as pseudogenes in the *P*. *knowlesi*.

We observed the antibodies against *P*. *vivax* antigens showed the expected localization pattern for the surface or apical organelles of *P*. *knowlesi* based on the localization information on *P*. *vivax*. Complete surface colocalization was observed with the anti-PvMSP1-19 antibody in *P*. *knowlesi*, consistent with the 82.6% amino acid identities of PvMSP1-19 to orthologs of *P*. *knowlesi* MSP1-19 and suggestive to a previous report [21]. Although syntenic orthologs of PvRBP1a and PvRBP1b do not exist in *P*. *knowlesi*, these antibodies reacted to the apical side of *P*. *knowlesi* merozoites, suggesting that non-syntenic paralogues were recognized, PkNBPXa or PkNBPXb. Protein similarities may explain this phenomenon as similar protein sequences do not always produce similar protein structures [58,59]. Previous studies supported that the structural similarities observed among *P*. *falciparum* Erythrocyte membrane protein 1 variants, PvDBP and Variant surface antigen 2-CSA, and PvAMA1 and PfAMA1 were shown to mediate cross-reactivity to a conserved epitope [60–62].

The functional activity of *P*. *vivax*-specific antibodies against heterologous parasites was also evaluated to determine whether antibodies could inhibit *P*. *knowlesi* invasion. We found that compared with controls, most of the *P*. *vivax*-specific antibodies could effectively block the invasion of *P*. *knowlesi*. Among all the antibodies, the anti-Pv41, anti-PvRBP1a, and anti-PvRhopH2 antibodies appeared to have the highest growth inhibition assay, which is in line with the recognition of either surface or apical organelles of the merozoite by these antibodies. Interestingly, anti-PvAMA1 and anti-PvMSP1-19 antibodies, which also have strong recognition of native and recombinant proteins, did not show high inhibitory activity against human erythrocyte invasion by *P*. *knowlesi*. This phenomenon was also observed in a previous study [61] in which an anti-PvAMA1 antibody did not inhibit *P*. *falciparum*, even though immunoassays showed high cross-reactivity to merozoites, and highlights the need for functional characterization of inhibitory activity. Despite the similarity in protein structure, the difference in molecules charges in the MSP1-19 domain results in no functional antibody activity [36]. The small change in the sequence may lead to a change in epitope recognition by changing the affinity and avidity of the antibody due to the loss of the inhibitory effect of epitope-antibody binding [63,64]. On the other hand, this study has limitation that we were not able to perform the reverse-growth inhibition assay using *P*. *knowlesi* blood-stage antibodies against *P*. *vivax* parasite, because of lack of long-term in vitro culture system of *P*. *vivax* parasites. Moreover, the further studies are required to reinforce the cross-species reactivity study.

One of the critical issues of the current malaria vaccine development is the low efficacy, for example, RTS,S vaccine showed only around 30% efficacy against children aged from 6–12 months after administration [65]. Such low protective efficacy would be improved if multiple antigens are used. As compared to an antibody specific for the single antigen, pooled antibodies for three antigens showed the additive effect to inhibit *P*. *knowlesi* invasion into human erythrocytes. The selection of these antibodies may play a role in this observation, as it was proposed that antibodies against merozoite surface slowed down the invasion to allow antibodies against proteins secreted from microneme and rhoptry to bind their targets [66,67].

Southeast Asia is heavily endemic for *P*. *vivax*, and recently *P*. *knowlesi* is also recognized to cause zoonotic human malaria in some regions in this area. Thus it is of interest to know if antibodies against one species could play a role in the cross-protection against other *Plasmodium* species. To this end, we found human IgG against *P*. *vivax* antigens is able to block the erythrocyte invasion by *P*. *knowlesi*. Failure to inhibit *P*. *knowlesi* invasion with anti-PvRBP1a IgG maybe because the epitopes recognized by this antibody were not protective epitopes of PvRBP1a homologs in other *Plasmodium* species. However, the limitation of this study is that we could not purify the knowlesi specific antibodies to perform the invasion inhibition assay because we did not have enough *P*. *knowlesi*-infected patients’ serum at that time.

As part of the importance of cross-reactivity, the immunity produced against other species could be one of the pivotal factors affecting the dynamic change in parasite-parasite interactions. Previous studies have shown that serological cross-reactivity is observed between *P*. *falciparum* and *P*. *vivax* and between *P*. *vivax* and *P*. *knowlesi* [25,68]. In this study, three different reactivities were observed for pooled *P*. *knowlesi* and/or *P*. *vivax* patients serum against *P*. *vivax* and/or *P*. *knowlesi* antigens; (1) *P*. *vivax* patients sera are more reactive than *P*. *knowlesi* patients sera to *P*. *vivax* antigens and vice versa, which is expected, (2) *P*. *knowlesi* patients sera are more reactive than *P*. *vivax* patients sera to *P*. *vivax* antigens and vice versa, which may be interpreted that cross-reacting epitopes exist not only in their closest orthologs but also in other members encoded by a multigene family [60] so that the antibody binding sites recognize the unrelated proteins [69], and (3) similar reactivity in both patients sera, which suggests shared common epitopes with the closest orthologs. Surprisingly, this study showed that approximately half of the human clinical patients from endemic area are cross-reactive to *P*. *knowlesi* MSP1-42, MAS180-N or AMA1. These results are important evidence indicating that immunity established against one *Plasmodium* species is able to cross-react against other *Plasmodium* species through shared common epitopes. However, the regular exposure to different *Plasmodium* species in one particular area may be correlated with the high reactivity of individual patient serum as it might lead to the accumulation of cross-immune antibodies. It is also noted that some of the *P*. *vivax*-infected patients possess seropositive to either single *P*. *knowlesi* antigen or in combination, highlighting the cross-protection antibodies for which some patients in the endemic area develop less severe symptoms or low parasitemia [70].

In the malaria pre-elimination era, the emergence of *P*. *knowlesi* and other zoonotic *Plasmodium* species are one of the obstacles to successfully control malaria in Southeast Asian countries. Cross-species invasion inhibition activities of antibodies against *P*. *vivax* shown in this study provides important platform to design multi-component malaria vaccines and suggesting the cross-species immunity affecting the disease dynamic change in Malaysia or endemic area. Some patients in malaria-endemic area might possess different antibodies against different *Plasmodium* species. However, the longevity of the cross-immune antibody is still questioned, whether or not it can be maintained long enough to give a cross-protection when the transmission change.

## Supporting information

S1 Fig Schematic diagrams of *P*. *vivax* blood-stage antigens with their homologs in *P*. *knowlesi*. Regions used to raise antibodies are shown in brown boxes with the first and the last amino acid positions at the top of diagram. The characteristic of each target was labeled into different colors or patterns of the diagram with its amino acid position at the top. Four targets (MSP1, MSP1P, MSP8, and MSP10) were identified the EGF-domain at the C-terminal region. The characteristic of each target was obtained from www.plasmodb.org.
(TIF)

S2 Fig Cross-species reactivity of *P*. *vivax*-specific antibodies with *P*. *knowlesi* parasites assessed by western blot analysis. *P*. *vivax*-specific antibodies recognized *P*. *knowlesi* parasite lysates. R, uninfected RBCs; P, *P*. *knowlesi* parasite lysate. The predicted full-length proteins were shown in green, and the possible processing-protein products were indicated with red arrowheads.
(TIF)

S3 Fig The dose dependent growth inhibition activity of *P*. *vivax* and *P*. *knowlesi* antibodies. Growth inhibition activity was evaluated using four different concentration (0.5; 1.0; 1.5 and 2.0/mL) rabbit IgG. The non immunized rabbit IgG and 2C3 monoclonal antibody were served as controls. The dose dependent inhibition of activity of PkDBPα and PkAMA1 antibodies were published online [47].
(TIF)

S4 Fig Additive effect of combined antibodies against *P*. *vivax* proteins with different localization to *P*. *knowlesi*. Growth inhibition activity was evaluated using 0.6 mg/mL rabbit IgG as a single antibody or in combination of 3 antibodies at final 2 mg/mL. The single and combination antibody was compared using one-way ANOVA with Dunnett’s test. *** = *p* value < 0.001.
(TIF)

S5 Fig The human antibody response to PvRBP1a-F (folded) and PvRBP1a-UF (unfolded) recombinant proteins. The prevalence of seropositivity was compared between patients (P: *P*. *vivax-*infected patients) and healthy individual (H) using the Mann-Whitney test. * = *p* value < 0.05; ns = non significant.
(TIF)

S6 Fig Western blot analysis of protein expression using the wheat germ cell-free analysis. The expression level of each protein was detected by using anti-His tag antibody. Total fraction (T) was then centrifuged to separate the soluble fraction (S) and pellet. A total 10 μL of proteins were loaded in each well. His-tag antibody was used to confirm the positive band in western blotting. The specific band that appears in the total fraction (T) was then said as non-soluble protein. Otherwise soluble protein was showed in a specific band appear in both total fraction and soluble fraction by western blotting.
(TIF)

S7 Fig Heatmap of antibody response in each individual patient to the three different antigens. (A) Antibody response to the three antigens in individual *P*. *knowlesi*-infected patients. (B) Antibody response to the three antigens in individual *P*. *vivax*-infected patients. Hierarchical clustering of individual patients reactivity was showed by a vertical dendrogram. The cluster of antibody reactivity in each patient for the three antigens was created based on the Euclidean distance. The red asterisk indicates the same individual with all three seropositive antigens. The color gradient (from blue to red) indicates the adjusted MFI value of antibody reactivity.
(TIF)

S1 Table The colocalization of *P*. *vivax* antibodies and *P*. *knowlesi* on IFA. (DOCX)

S2 Table Summary of cloning primers for *P*. *knowlesi* recombinant protein expression. (DOCX)

S3 Table Summary of *P*. *vivax* antibodies used in this study. (DOCX)
