# Supplementary figures and images for: Cross-species reactivity of antibodies against Plasmodium vivax blood-stage antigens to Plasmodium knowlesi
Source: PLoS Negl Trop Dis. 2020 Jun 19;14(6):e0008323. doi: 10.1371/journal.pntd.0008323 (PMC7304578; doi:10.1371/journal.pntd.0008323)

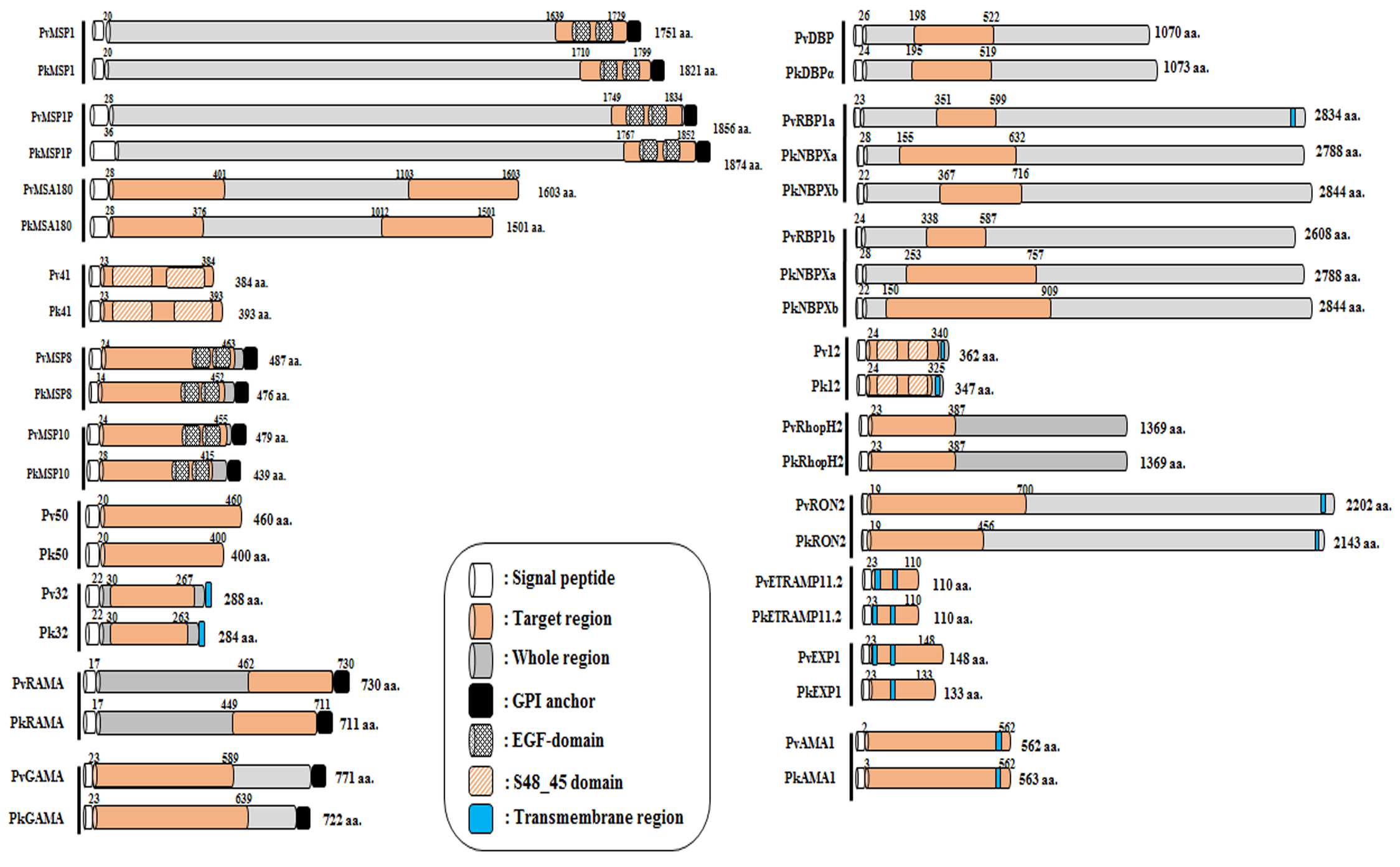

Supplement: S1 Fig — Regions used to raise antibodies are shown in brown boxes with the first and the last amino acid positions at the top of diagram. The characteristic of each target was labeled into different colors or patterns of the diagram with its amino acid position at the top. Four targets (MSP1, MSP1P, MSP8, and MSP10) were identified the EGF-domain at the C-terminal region. The characteristic of each target was obtained from www.plasmodb.org. (TIF) [file pntd.0008323.s001.tif]

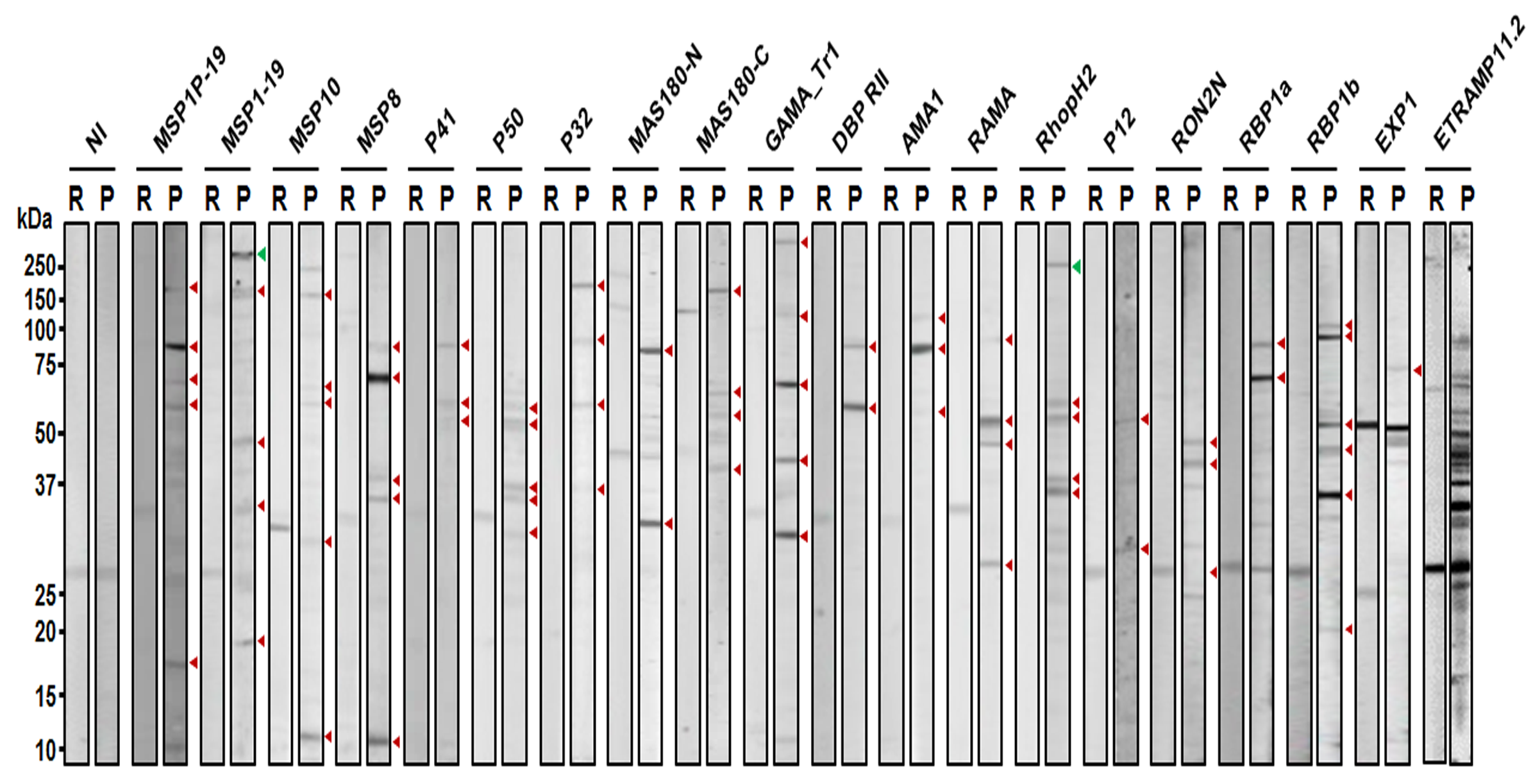

Supplement: S2 Fig — P. vivax-specific antibodies recognized P. knowlesi parasite lysates. R, uninfected RBCs; P, P. knowlesi parasite lysate. The predicted full-length proteins were shown in green, and the possible processing-protein products were indicated with red arrowheads. (TIF) [file pntd.0008323.s002.tif]

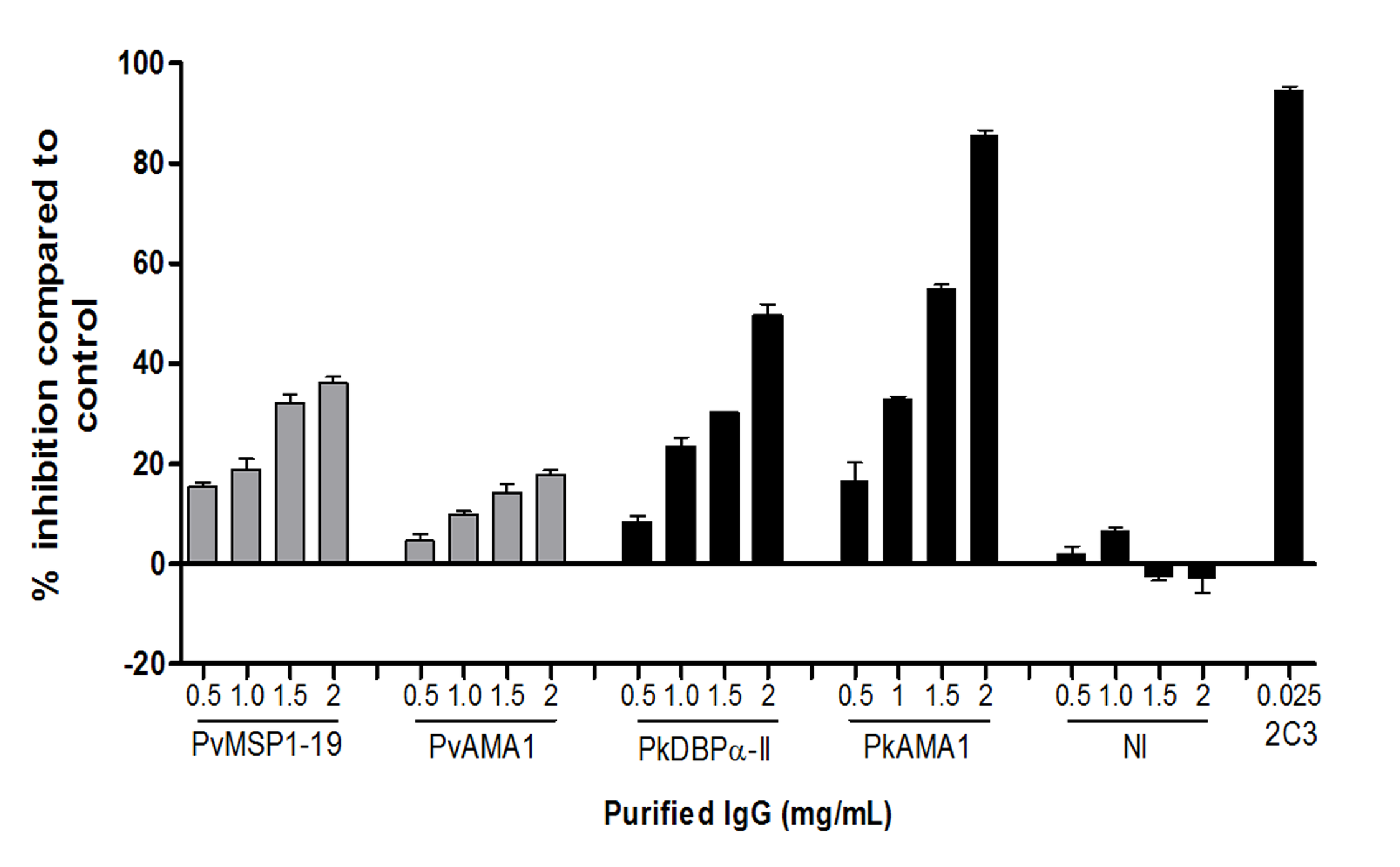

Supplement: S3 Fig — Growth inhibition activity was evaluated using four different concentration (0.5; 1.0; 1.5 and 2.0/mL) rabbit IgG. The non immunized rabbit IgG and 2C3 monoclonal antibody were served as controls. The dose dependent inhibition of activity of PkDBPα and PkAMA1 antibodies were published online [47]. (TIF) [file pntd.0008323.s003.tif]

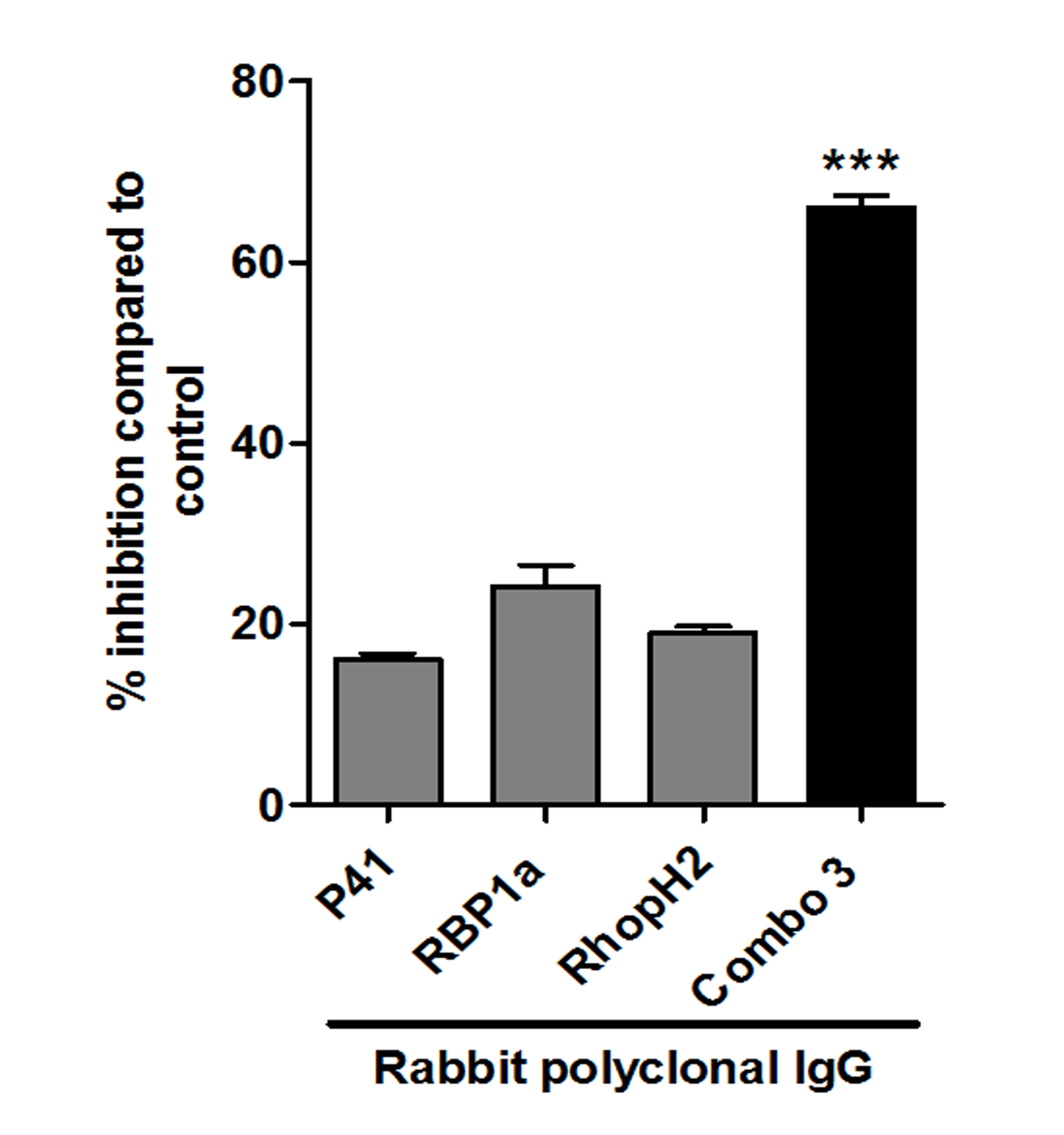

Supplement: S4 Fig — Growth inhibition activity was evaluated using 0.6 mg/mL rabbit IgG as a single antibody or in combination of 3 antibodies at final 2 mg/mL. The single and combination antibody was compared using one-way ANOVA with Dunnett’s test. *** = p value < 0.001. (TIF) [file pntd.0008323.s004.tif]

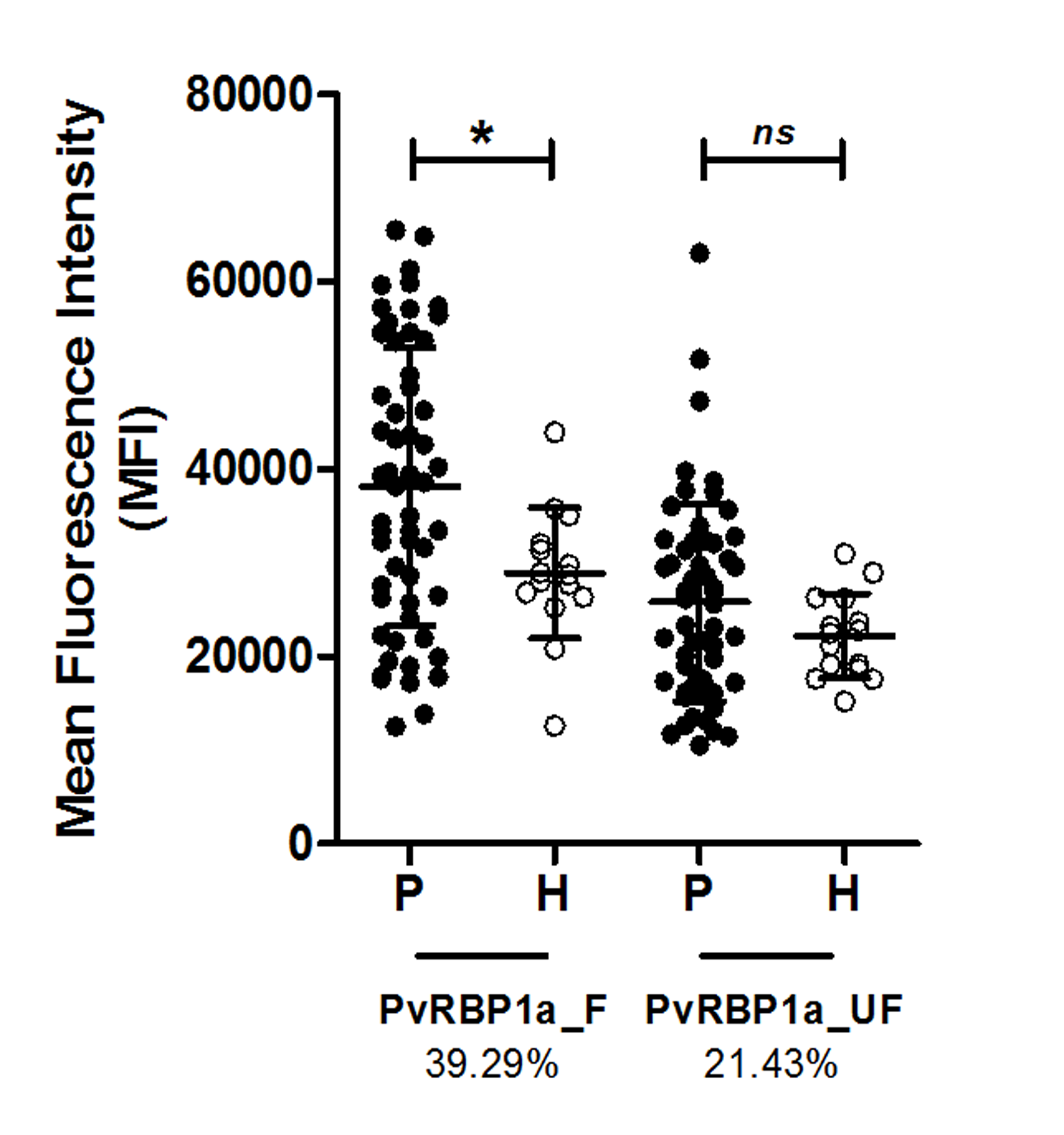

Supplement: S5 Fig — The prevalence of seropositivity was compared between patients (P: P. vivax-infected patients) and healthy individual (H) using the Mann-Whitney test. * = p value < 0.05; ns = non significant. (TIF) [file pntd.0008323.s005.tif]

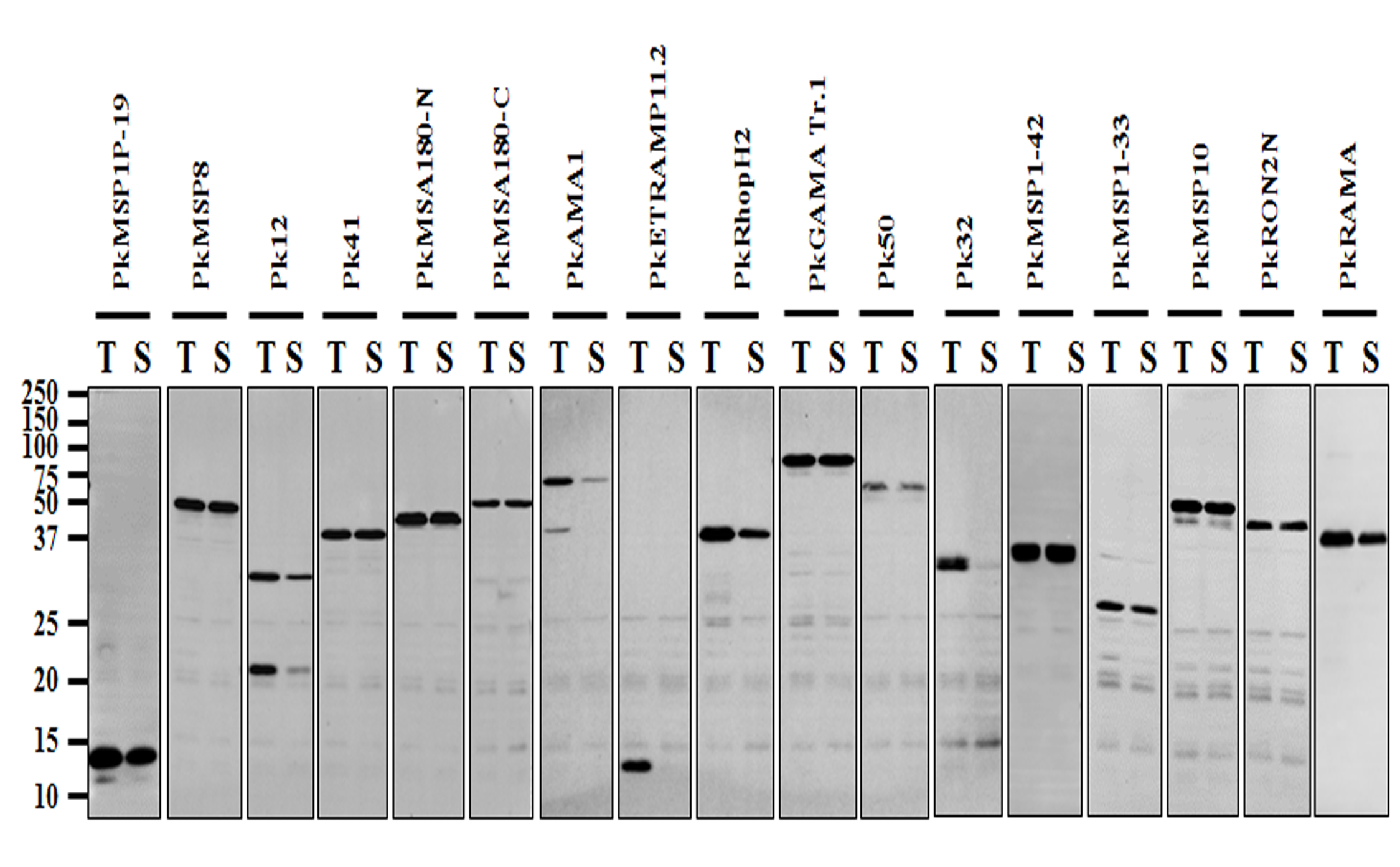

Supplement: S6 Fig — The expression level of each protein was detected by using anti-His tag antibody. Total fraction (T) was then centrifuged to separate the soluble fraction (S) and pellet. A total 10 μL of proteins were loaded in each well. His-tag antibody was used to confirm the positive band in western blotting. The specific band that appears in the total fraction (T) was then said as non-soluble protein. Otherwise soluble protein was showed in a specific band appear in both total fraction and soluble fraction by western blotting. (TIF) [file pntd.0008323.s006.tif]

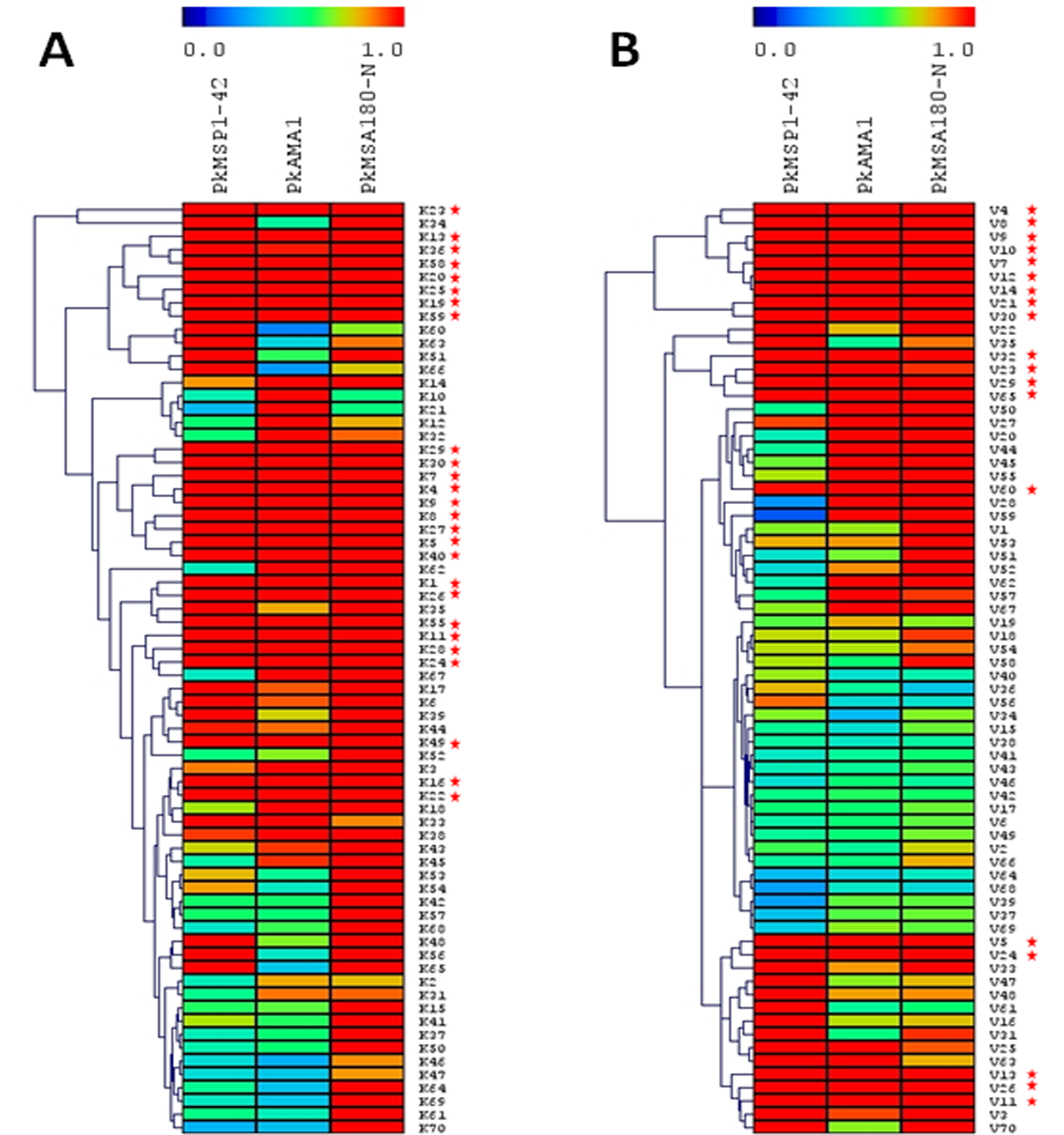

Supplement: S7 Fig — (A) Antibody response to the three antigens in individual P. knowlesi-infected patients. (B) Antibody response to the three antigens in individual P. vivax-infected patients. Hierarchical clustering of individual patients reactivity was showed by a vertical dendrogram. The cluster of antibody reactivity in each patient for the three antigens was created based on the Euclidean distance. The red asterisk indicates the same individual with all three seropositive antigens. The color gradient (from blue to red) indicates the adjusted MFI value of antibody reactivity. (TIF) [file pntd.0008323.s007.tif]
