# Supplementary material for: Cross-species reactivity of antibodies against Plasmodium vivax blood-stage antigens to Plasmodium knowlesi
Source: PLoS Negl Trop Dis. 2020 Jun 19;14(6):e0008323. doi: 10.1371/journal.pntd.0008323 (PMC7304578; doi:10.1371/journal.pntd.0008323)
Supplement: S1 Table — (DOCX) [file pntd.0008323.s008.docx]

**S1 Table. The colocalization of *P. vivax* antibodies and *P. knowlesi* on IFA.**

| **Blood-stage antibodies** | **Colocalization (*r^2^* value)** |
| --- | --- |
| PvMSP1P-19 | 76.2 |
| PvMSP1-19 | 97.3 |
| PvMSP10 | 76.5 |
| PvMSP8 | 38.0 |
| Pv41 | 86.3 |
| Pv50 | 79.8 |
| Pv32 | 91.3 |
| PvMSA180-N | 97.0 |
| PvMSA180-C | 85.0 |
| PvGAMA_Tr1 | 77.7 |
| PvDBP-RII | 27.2 |
| PvAMA1 | 72.4 |
| PvRBP1a | 84.4 |
| PvRBP1b | 85.2 |
| Pv12 | 70.3 |
| PvRON2N | 36.5 |
| PvRAMA | 43.4 |
| PvRhopH2 | 76.8 |
| PvETRAMP 11.2 | 27.3 |
| PvEXP1 | 42.1 |
