# Supplementary material for: Cross-species reactivity of antibodies against Plasmodium vivax blood-stage antigens to Plasmodium knowlesi
Source: PLoS Negl Trop Dis. 2020 Jun 19;14(6):e0008323. doi: 10.1371/journal.pntd.0008323 (PMC7304578; doi:10.1371/journal.pntd.0008323)
Supplement: S2 Table — (DOCX) [file pntd.0008323.s009.docx]

**S2 Table. Summary of cloning primers for *P. knowlesi* recombinant protein expression**

| **Gene ID** | **Name** | **Forward primer (5'→3')^a^** | **Reverse primer (5'→3')^a^** |
| --- | --- | --- | --- |
| PKNH_0728800 | MSP1P-19 | gggcggatatctcgagGATCGTGTGAAAAATAACTGCAGA | gcggtacccgggatccttaGCACACGACCCCCTCATATA |
| PKNH_0728900 | MSP1-19 | gggcggatatctcgagTTAAATATGAGTTCCGCACATAAGTG | gcggtacccgggatccttaGCAGGCTGCATTTTCAGGTAC |
| PKNH_0728900 | MSP1-33 | gggcggatatctcgagGAGAATCACGTGGCTGCATTC | gcggtacccgggatccttaGCAGGCTGCATTTTCAGGTAC |
| PKNH_0728900 | MSP1-42 | gggcggatatctcgagGAGAATCACGTGGCTGCATTC | gcggtacccgggatccttaGCAGGCTGCATTTTCAGGTAC |
| PKNH_1031500 | MSP8 | gggcggatatctcgagGGAAACGTCAACCCACCC | gcggtacccgggatccttaGCAGTATATTCCATCTCCTTGAAAG |
| PKNH_1129800 | MSP10 | gggcggatatctcgagGGGAATGAATTGAAAGGAACC | gcggtacccgggatccttaCAGGACACAGAAAATCCCATC |
| PKNH_0421000 | P32 | gggcggatatctcgagGCAGGAGGCGTTTCCGAA | gcggtacccgggatccttaCTTAGGGTTACAAAACAAGTCGGC |
| PKNH_0303000 | P41 | gggcggatatctcgagGAACACATCTGTGATTTCACCAA | gcggtacccgggatccttaCTCATGGAAAGACTTAGCAACAAC |
| PKNH_0730000 | P50 | gggcggatatctcgagCATTTTTTCACCTTCTGTTCTGTG | gcggtacccgggatccttaTGCACTTCCTATTATCTCCGTCT |
| PKNH_0814000 | MSA180-N | gggcggatatctcgagGATGATGACAAAAATAAAAGGGCT | gcggtacccgggatccttaACCGCCATAGTAAATGTTGTCAT |
| PKNH_0814000 | MSA180-C | gggcggatatctcgagTGCATCGGTGGATGTAGC | gcggtacccgggatccttaCGCTTCCTCGTTGATGTG |
| PKNH_0931500 | AMA1 | gggcggatatctcgagAAAATATACTACATACTATTTTTAAGCGCT | gcggtacccgggatccttaGTAGTAAGGCTTCTCCATCAGAAC |
| PKNH_1322900 | GAMA_Tr1 | gggcggatatctcgagCGAAATGGGAACAATCCG | gcggtacccgggatccttaTCCGCTTCCGTTGACTTC |
| PKNH_1137300 | P12 | gggcggatatctcgagTTCACACACACATGTGATTTCAATG | gcggtacccgggatccttaTGCACCACTGCGACCCTG |
| PKNH_1230100 | RON2N | gggcggatatctcgagAGTACAAAAGAGGTAACAGGGAGC | gcggtacccgggatccttaTCCATTGCCATTTTTC |
| PKNH_0105800 | RAMA | gggcggatatctcgagAAGGAGGCTGTCAAGAAAGGA | gcggtacccgggatccttaACTAGTGAGACATAGTAATCCGATCAG |
| PKNH_0727900 | RhopH2 | gggcggatatctcgagGAGTTAGGCCATACCGTGTCC | gcggtacccgggatccttaCTTCTCGATGTCTTCGTAGTCCA |
| PKNH_1137300 | ETRAMP 11.2 | gggcggatatctcgagTTCTACAATAATGTTGTAGAGGGAAAG | gcggtacccgggatccttaTTGGGTTCTGGTGGTGGT |

^a^ Lowercase latter of primer sequence indicates it is homologous to the vector sequence for in-fusion reaction.
