# Supplementary material for: Cross-species reactivity of antibodies against Plasmodium vivax blood-stage antigens to Plasmodium knowlesi
Source: PLoS Negl Trop Dis. 2020 Jun 19;14(6):e0008323. doi: 10.1371/journal.pntd.0008323 (PMC7304578; doi:10.1371/journal.pntd.0008323)
Supplement: S3 Table — (DOCX) [file pntd.0008323.s010.docx]

**S3 Table. Summary of *P. vivax* antibodies used in this study**

| **Name** | **PlasmoDB ID** | **Amino acid position (length)** | **Molecular weight (kDa)** | **Expression system** | **Protein tag** |
| --- | --- | --- | --- | --- | --- |
| PvMSP1P Sal I | PVX_099975 | 1749-1834 (86) | 9.6 | WGCF | His |
| PvMSP1 Sal I | PVX_099980 | 1639-1729 (91) | 9.8 | WGCF | His |
| PvMSP10 Sal I | PVX_114145 | 24-455 (432) | 47.0 | WGCF | His |
| PvMSP8 Sal I | PVX_097625 | 24-463 (440) | 49.3 | WGCF | His |
| Pv41 Sal I | PVX_000995 | 23-384 (362) | 41.7 | WGCF | His |
| Pv50 Sal I | PVX_087140 | 20-460 ((441) | 48.4 | WGCF | His |
| Pv32 Sal I | PVX_084815 | 30-267 (238) | 26.7 | WGCF | GST |
| PvMSA180 Sal I | PVX_094920 | 28-401 (374)  N-term  1103-1603 (501) C-term | 43.0 (N-term)  57.9 (C-term) | *E. coli* | N-term (GST)  C-term (His) |
| PvGAMA Sal I | PVX_088910 | 23-589 (567) | 57.5 | WGCF | His |
| PvDBP Sal I | PVX_110810 | 198-522 (325) | 38.5 | *E. coli* | His |
| PvAMA1 Sal I | PVX_092275 | 2-562 (561) | 64.5 | WGCF | His |
| PvRBP1a Sal I | PVX_098585 | 351-599 (249) | 34.4 | WGCF | His |
| PvRBP1b Sal I | PVX_098582 | 338-587 (250) | 32.0 | WGCF | His |
| Pv12 Sal I | PVX_113775 | 24-340 (317) | 36.0 | WGCF | His |
| PvRON2 Sal I | PVX_117880 | 19-700 (682) | 68.9 | WGCF | His |
| PvRAMA Sal I | PVX_087885 | 462-730 (269) | 27.9 | WGCF | His |
| PvRhopH2 Sal I | PVX_099930 | 23-387 (365) | 42.6 | WGCF | His |
| PvETRAMP 11.2 Sal I | PVX_003565 | 23-110 (88) | 9.5 | WGCF | His |
| PvEXP1 Sal I | PVX_091700 | 23-148 (126) | 9.6 | WGCF | His |

WGCF, Wheat germ cell-free system.
